# Generalized Latent Variable Models for Location, Scale, and Shape parameters

**DOI:** 10.1017/psy.2025.7

**Published:** 2025-03-06

**Authors:** Camilo A. Cárdenas-Hurtado, Irini Moustaki, Yunxiao Chen, Giampiero Marra

**Affiliations:** 1 Department of Statistics, The London School of Economics and Political Science, London, UK; 2 Department of Statistical Science, University College London, London, UK

**Keywords:** distributional regression, EM algorithm, GAMLSS, heteroscedasticity, latent variable models

## Abstract

We introduce a general framework for latent variable modeling, named Generalized Latent Variable Models for Location, Scale, and Shape parameters (GLVM-LSS). This framework extends the generalized linear latent variable model beyond the exponential family distributional assumption and enables the modeling of distributional parameters other than the mean (location parameter), such as scale and shape parameters, as functions of latent variables. Model parameters are estimated via maximum likelihood. We present two real-world applications on public opinion research and educational testing, and evaluate the model’s performance in terms of parameter recovery through extensive simulation studies. Our results suggest that the GLVM-LSS is a valuable tool in applications where modeling higher-order moments of the observed variables through latent variables is of substantive interest. The proposed model is implemented in the R package glvmlss, available online.

## 1 Introduction

Latent variable models (LVMs) are widely used in the social sciences to measure unobserved constructs of interest using several correlated observed variables. The Generalized Linear LVM (GLLVM, Bartholomew et al., 2011; Moustaki & Knott, 2000; Skrondal & Rabe-Hesketh, 2004) is a versatile modeling framework where (i) conditional on the latent variables, each observed variable follows a distribution from the exponential family, and (ii) only the mean of this conditional distribution depends on the latent variables. The GLLVM encompasses various LVMs for continuous, categorical, and count observed variables, making it a widely used tool for analyzing multivariate data.

While the GLLVM framework is flexible, it can sometimes oversimplify real-world scenarios by relying only on distributions from the exponential family. In specific applications, it is essential to model higher-order moments of the observed variables, such as variance, skewness, and kurtosis, as functions of the latent variables. Ignoring these features of the observed data can result in underestimated standard errors (SEs), biased parameter estimates, and inaccurate model fit indices (Lai, 2018; Lei & Lomax, 2005; Wall et al., 2015).

The challenges above have been extensively studied in the literature. Limited information and robust estimation methods have been proposed to address deviations from distributional assumptions (e.g., Bollen, 1996; Browne, 1984; Moustaki & Victoria-Feser, 2006). Furthermore, new advanced models have been developed, such as the heteroscedastic factor models (e.g., Hessen & Dolan, 2009; Lewin-Koh & Amemiya, 2003), LVMs for continuous data displaying skewness and/or kurtosis (e.g., Asparouhov & Muthén, 2016; Liu & Lin, 2015; Molenaar et al., 2010; Montanari & Viroli, 2010), factor models for discrete, count, and bounded continuous data with zero/one/maximum inflation and heaping (e.g., Magnus & Thissen, 2017; Molenaar et al., 2022; Niku et al., 2017; Wall et al., 2015; Wang, 2010), and LVMs for censored/truncated data (e.g., Moustaki & Steele, 2005). However, these models have primarily been developed in isolation and differ in their estimation and inferential methods.

This article introduces a comprehensive modeling framework called the Generalized LVM for Location, Scale, and Shape parameters (GLVM-LSS). This framework models the conditional distribution of each observed variable as a function of latent variables. We achieve this by defining the distributional parameters characterizing each observed variable’s conditional distribution as functions of the latent variables. Since the mean and other higher-order moments of the observed variables are expressed in terms of their distributional parameters, they also depend on the latent variables.

The GLVM-LSS borrows ideas from the Generalized Additive Model for Location, Scale, and Shape (GAMLSS) regression framework (Klein et al., 2015; Rigby & Stasinopoulos, 2005; Umlauf et al., 2018), and applies them to models with latent variables. The GAMLSS is a flexible regression framework where observed covariates have linear or nonlinear effects on the distributional parameters characterizing the distribution of the outcome variable. It can also accommodate spatial, temporal, and random effects. For a comprehensive treatment of the GAMLSS regression framework, see, e.g., Stasinopoulos et al. (2017, 2024), and Rigby et al. (2020). In this article, we only consider *linear* effects of the latent variables on the distributional parameters.

Our proposed framework shares similarities with previous works in the LVM literature. For example, in multilevel or longitudinal studies (Hedeker & Gibbons, 2006; Skrondal & Rabe-Hesketh, 2004), location-scale mixed-effects models accommodate covariates and random effects on both location and scale parameters for continuous (Hedeker et al., 2008, 2012) and binary/ordered categorical (Greene, 2003; Hedeker et al., 2006, 2009, 2016) observed variables (the latter using an underlying response formulation, see Remark 2). These models can be fitted using commercial software like the gllamm module in Stata (Rabe-Hesketh et al., 2004) or the PROC NLMIXED routine in SAS. An important remark is that multiple group LVMs (Davidov et al., 2018) also allow for different (conditional) means and (conditional) variances among the groups.

### 1.1 Motivating examples

We present two motivating examples from different fields where modeling higher-order moments of complex multivariate datasets is of substantive interest. These examples are discussed further in Section 3.

Example 1. The first example comes from public opinion research, where we examine people’s attitudes toward different social groups using survey data from the 2020 American National Election Study (ANES 2020). Respondents rate their feelings about these groups on a scale from 0 to 100, with higher ratings indicating more favorable opinions. Although the questions are not explicitly designed to measure a particular latent construct, they provide insight into respondents’ positions on a *conservative–progressive* belief scale. We could model the conditional mean of these doubly-bounded responses as a function of the latent variable using a Beta factor model (Noel, 2014; Noel & Dauvier, 2007; Revuelta et al., 2022). However, research has shown that liberals are more likely to have similar political attitudes compared to conservatives (Ondish & Stern, 2018), suggesting that the *conservative–progressive* latent factor influences not only the conditional mean but also the conditional variance of the responses. Addressing this issue is possible with a *heteroscedastic* Beta factor model, which we introduce in Section 2.1. Most work on factor models for continuous doubly-bounded observed variables focuses on modeling only the location parameter (conditional mean) in terms of the latent variables. The scale parameter related to the conditional variance does not depend on the latent variables. A notable exception is the mixed and mixture Beta regression model by Verkuilen & Smithson (2012), where the scale parameter of the Beta distribution is modeled using random effects.

Example 2. The second example comes from educational testing, using data from the PISA 2018 computer-based mathematics exam. We present a confirmatory factor model to analyze binary item responses (IRs) and continuous response times (RTs) simultaneously. van der Linden (2007, 2009) proposed a joint model in which the two-parameter logistic model is applied to the IRs, while a linear factor model is used for the log-RTs. To account for the “speed-accuracy trade-off” in educational testing (Zimmerman, 2011), it is assumed that the latent ability and speed factors are correlated. However, van der Linden’s model for the log-RTs (conditional mean) can be restrictive because RTs tend to have a variance that increases with the mean (De Boeck & Jeon, 2019; Van Zandt, 2002). Specifically, the variance parameter, which helps discriminate between test takers with different speed levels, does not depend on the respondent’s latent speed trait. Furthermore, modeling additional aspects of the RT distribution as a function of the latent speed trait, such as the (conditional) variance and (conditional) skewness, can provide further insights into individuals’ test-taking strategies and items’ characteristics. In Section 2.1, we present a joint model for IRs and RTs in which log-RTs follow a Skew-Normal distribution (SN, Azzalini, 1985, 2005), with distributional parameters influencing higher order moments modeled as functions of the individual’s latent speed trait. Although alternative parametric distributions have been used to model RTs in the hierarchical model framework (e.g., Loeys et al., 2011), the focus is still on the location parameter.

The article is organized as follows. In Section 2, we introduce the GLVM-LSS model, discuss parameter estimation via full-information marginal maximum likelihood (MML) estimation, and examine model identification. In Section 3, we apply the proposed method to real-world data on public opinion research and educational testing. To demonstrate the properties of our proposed method under finite sample settings, we conduct simulation studies in Section 4. Finally, we discuss some limitations and future research directions.

## 2 GLVM-LSS

### 2.1 Proposed model framework

Let 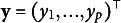 be a random vector of observed variables with domain 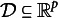 , and 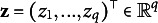 a random vector of continuous latent variables, with *q* (much) smaller than *p*. Assuming local independence (Bartholomew et al., 2011, Chapter 1), the marginal distribution of 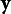 is: (1)

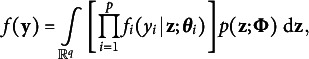

where, for observed variables 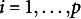 , 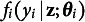 is the conditional distribution of 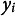 given 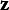 and 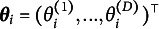 is a *D*-dimensional vector of distributional parameters indexing 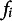 . The (conditional) distributional moments of 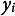 (mean, variance, skewness) are functions of the parameters 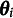 . 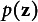 is the prior distribution of 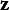 , commonly assumed to be a multivariate Normal distribution with covariance matrix 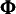 , 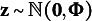 .

We propose a class of GLVM-LSS where the distributional parameters 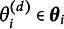 are expressed as monotone functions of linear combinations of the latent variables. We write 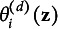 to denote the functional dependence of the distributional parameter on 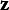 , but in most cases we omit it to simplify notation. Moreover, we use the sub-index 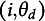 to indicate that the corresponding function or model parameter is related to 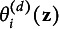 . The relationship between 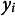 and 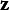 is therefore through the vector 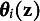 in 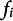 and is determined by the system of equations: (2)

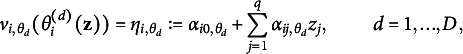

where the parameter-specific link function, denoted by 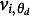 , is used to ensure that the distributional parameters have appropriate restrictions. The link function can be identity, log, logit, or any other suitable monotone function. 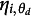 represents the linear combination of latent variables, with intercept 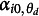 and factor loadings grouped in the *q*-dimensional vector 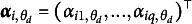 .

The distributional parameters 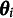 that describe the shape of 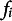 can be divided into three categories: location, scale, or shape parameters. Their role depends on which distributional moment of 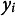 they define. To simplify notation, we refer to the location parameter as 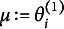 , the scale parameter as 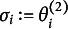 , and the shape parameters as 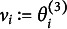 and 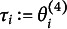 . In most cases, a maximum of four parameters (one location, one scale, and two shape parameters) is enough. However, this framework can be extended to include distributions with multiple location, scale, or shape parameters. We then write 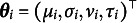 to denote the vector of distributional parameters indexing 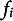 , and use 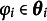 to refer to any location, scale, or shape parameter in the (conditional) distribution of 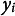 .

For a more compact matrix notation, let 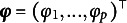 be the vector of the same distributional parameter 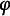 for all 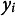 ’s. Denote 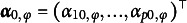 as a vector of intercepts, and let 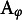 be a 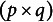 factor loadings matrix with rows corresponding to the vectors 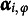 . Finally, let 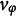 be the vector function that applies the corresponding link function 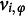 to each entry of 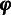 . Under this convention, the set of equations for a distributional parameter 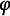 is 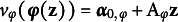 .

To further simplify notation, we write the vector of parameters 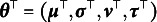 , the vector of intercepts 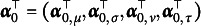 , and the factor loading matrix 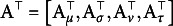 , to compactly express the system of equations of a GLVM-LSS model as: (3)

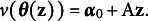

Remark 1. The GLVM-LSS framework is most useful when observed variables follow distributions with multiple location, scale and shape parameters, and it is appropriate and essential to model their higher-order moments as functions of the latent variables.

Remark 2. While standard LVMs for categorical data fit within the GLVM-LSS framework, accommodating models that involve scale parameters—such as the family of scaled (heteroscedastic) logistic or probit models for binary and ordinal data (see, e.g., Greene, 2003; Hedeker et al., 2006, 2009, 2016; Molenaar, 2015; Molenaar et al., 2012)—requires an alternative model specification. These models employ an *underlying variable* formulation (Jöreskog & Moustaki, 2001) for categorical variables where the observed category denoted by 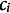 of the ordinal manifest variable 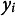 is determined by an unobserved *continuous* response 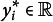 underlying the ordinal variable 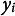 . The connection between 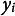 and 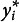 is 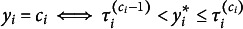 , where 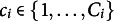 and 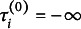 , 
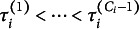
 , 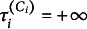 are called threshold parameters. Here, 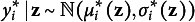 , and appropriate measurement equations for the location and scale parameters of the underlying response are chosen. Binary variables are special cases. In principle, the underlying response formulation for binary and ordered categorical data can be accommodated within the GLVM-LSS framework. However, the current proposed GLVM-LSS framework includes only the homoscedastic logit model. We plan to incorporate the heteroscedastic logit/probit model in future work on the GLVM-LSS framework.

Remark 3. Additional attention is required for discrete observed variables following distributions with distributional parameters taking values in a discrete space (e.g., the natural numbers). In some cases, a reparametrization is available such that the distributional parameters are in the real numbers (see, e.g., Rigby et al. (2020, p. 483) for the Negative Binomial distribution case), and common modeling techniques can be used. However, if no alternative parametrization exists, these parameters should be treated as *fixed* and *known*. Otherwise, modeling these distributional parameters requires computational methods that are beyond the scope of this article (see, e.g., Choirat & Seri, 2012; Hammersley, 1950).

We now revisit the motivating examples in Section 1.1 to illustrate how the GLVM-LSS framework extends the existing LVM literature by modeling features of the observed data beyond the conditional mean.

Example 1 (continued). *A heteroscedastic Beta factor model:* We propose a novel heteroscedastic Beta factor model to model the conditional variance of the doubly-bounded variables in the 2020 ANES dataset. The observed variables conditional on the *conservative–progressive* latent variable (denoted by *z*) follow a location-scale reparametrization of the Beta distribution, 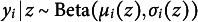 , where the location parameter 
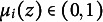
 and the scale parameter 
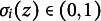
 are modeled as functions of *z* (Rigby et al., 2020, p. 461). Under this parametrization, we have 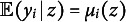 and 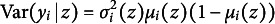 .

This parametrization is closely related to the location-precision parametrization in the heteroscedastic Beta regression literature (Smithson & Verkuilen, 2006; Verkuilen & Smithson, 2012), where a precision parameter 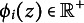 replaces the scale parameter, and 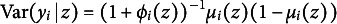 . Notably, 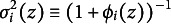 , meaning 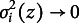 when 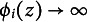 and 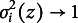 when 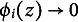 . However, the authors of the papers above report computational challenges and biased coefficient estimates in the precision parameter equation. Our empirical application results in Section 3.1 and the simulations study in Section 4.1 show that the location-scale parametrization used in this article avoids these issues while enabling a direct interpretation of the effect of the latent variables on the conditional variance of the items.

The equations for the location and scale parameters are: (4)

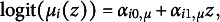


(5)

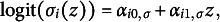

with the logit link function mapping the equations onto the correct space for the corresponding distributional parameter.

Example 2 (continued). *A confirmatory factor model for binary items and skewed RTs:* The GLVM-LSS specification of the joint model for IRs and responses times (RTs) is as follows. For IRs 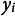 , 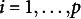 , we assume 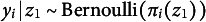 , where 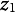 represents the student’s latent ability. The equations for the location parameters of the Bernoulli distribution are modeled as: (6)

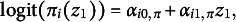

where 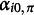 and 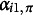 denote item *i*’s difficulty and discrimination parameters, respectively. For the RTs, we assume 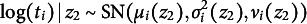 , with 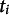 denoting item’s *i* RT in minutes and 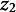 the student’s latent speed trait. Under this reparametrization of the SN distribution^1^, the equations for the location 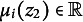 , scale 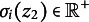 , and shape 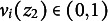 parameters are: (7)

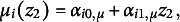


(8)

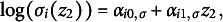


(9)

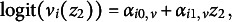

respectively, with the identity, log, and logit links mapping the equations onto the respective spaces of the distributional parameters. The (conditional) moments for the log-RTs are 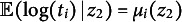 , 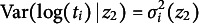 , and 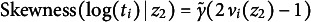 , with 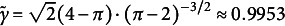 . Finally, to capture the “speed-accuracy trade-off,” the latent ability and speed traits are distributed as 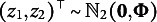 , where 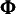 is a correlation matrix.

Remark 4. The proposed framework can be extended to accommodate linear and non-linear structural relationships between latent variables and/or observed covariates effects. Let 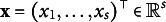 denote a vector of observed covariates. A linear structural model with covariate effects can be written in matrix form as: 

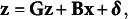

where 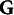 is a 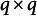 matrix of structural parameters satisfying recursive restrictions (Bollen, 1989), 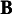 is a 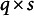 matrix of regression coefficients, and 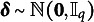 is a vector of independent standard Normal errors.

Similarly, assuming the vector of covariates 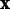 has a linear and additive effect on the linear component, the measurement equation for an arbitrary distributional parameter 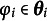 indexing 
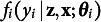
 becomes: (10)

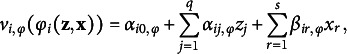

where 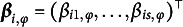 is a vector of regression coefficients. The measurement equation in (10) can also include interactions between the observed covariates and the latent variables to enable the study of “distributional” differential item functioning (DIF), where the assessment of DIF goes beyond the (conditional) mean and extends to the items’ (conditional) higher-order moments.

While Remark 4 highlights how the GLVM-LSS can be expanded into a more general LVM framework, this article aims to establish a foundation for future methodological developments in distributional LVM research. It should be noted, however, that the current implementation of the GLVM-LSS improves model fit over traditional approaches and proves valuable in empirical research where, from a measurement perspective, higher-order moments of observed variables carry substantive meaning or reflect important item characteristics.

For example, in the ANES 2020 application (Section 3.1), we examine whether individuals with liberal values have more homogeneous views on the social groups in question. If so, the (conditional) variance of the thermometer items should be lower for individuals on the *liberal* side of the latent scale than for those on the *conservative* side. In the PISA 2018 application (Section 3.2), modeling the scale (variance) and shape (skewness) parameter of the (log-)RTs as functions of the latent speed factor could help testing agencies better understand test-taking strategies and item characteristics.

### 2.2 Model identification

The generality of the proposed model makes it challenging to derive general conditions for global identification (e.g., Skrondal & Rabe-Hesketh, 2004, Chapter 5), so we instead resort to the weaker notion of (strict) local identification (Rothenberg, 1971).

As in the GLLVM, strict local identifiability in the GLVM-LSS is only possible for points on the *reduced* parameter space that results from imposing at least 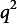 restrictions across the factor loadings matrix 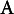 and the latent variables covariance matrix 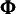 . These restrictions address rotational indeterminacy and fix the scale of the latent variable space (Anderson & Rubin, 1956). Denote by 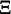 such *reduced* parameter space and 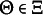 a point of free (unrestricted) model parameters. A point 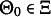 is strictly locally identifiable if the expected information matrix 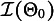 is strictly positive definite (Rothenberg, 1971). However, in the GLVM-LSS framework, model identifiability can be challenging even after imposing appropriate restrictions on the parameters due to the presence of multiple (possibly correlated) location, scale, and shape parameters. In what follows, we present theoretical results on the identifiability of GLVM-LSS models for continuous observed data following distributions with multiple distributional parameters.

Under suitable regularity conditions, we show that GLVM-LSS is generically locally identifiable in the sense that the model is strictly locally identifiable for almost all parameters in 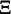 except for a subset with Lebesgue measure zero. More precisely, we define “generic local identifiability” as follows.

Definition 2.1. A statistical model is generically locally identified if every 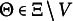 is (strictly) locally identifiable, where *V* is a proper sub-variety of 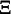 and thus has Lebesgue measure zero in 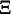 .

This notion of identifiability is closely related to and can be seen as a weaker version of the concept of “generic identifiability” (Allman et al., 2009; Gu & Xu, 2020) that has been commonly adopted for studying the identifiability of LVMs. The following theorem holds:

Theorem 2.1. Assume:

- There exists a point in the reduced parameter space 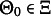 such that 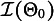 is strictly positive definite,

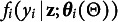
 , 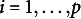 , in the measurement part, and 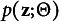 in the structural part of a GLVM-LSS model, are infinitely differentiable in 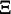 and 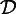 , and their respective supports are independent of 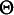 .

Then, the GLVM-LSS model is generically locally identified.

Assumption (A1) avoids trivial non-identification issues, and follows from the restrictions imposed to solve the rotational and scale indeterminacies. This assumption also rules out cases where the distributional parameters in 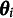 are linearly dependent. Assumption (A2) relates to smoothness and regularity conditions often met in practice for continuous distributions indexed by multiple location, scale, and shape parameters. The proof of Theorem 2.1, and auxiliary definitions, lemmas, and propositions are provided in the Section A3 of the Supplementary Material.

While Theorem 2.1 addresses models with continuous observed data, establishing general identification conditions for GLVM-LSS models with categorical data are more challenging due to the finite amount of information in the data. In these cases, parameter identification follows from the existence of a finite-dimensional sufficient statistic. Indeed, once appropriate parameter restrictions are imposed, a necessary condition for a GLVM-LSS with categorical items to be identified is that the number of parameters is less than the number of possible response patterns (e.g., 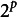 for binary items or 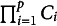 for categorical items, where 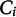 is the number of categories for item *i*). We note that, as mentioned in Remark 2, the current specification of the proposed framework does not cover heteroscedastic models for binary/ordinal categorical data and therefore the identification constraints described in, e.g., Skrondal and Rabe-Hesketh (2004, Chapter 2), Hedeker et al. (2006, 2009) or Molenaar (2015), Molenaar et al. (2012), do not apply to the current setting.

In practice, some 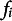 ’s might be indexed by distributional parameters that are correlated (yet linearly independent). Due to sampling variability, the latter can lead to situations where the model is not empirically identified. In this case, empirical local identification of the MLE can be verified if the estimated expected information matrix, 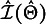 , is non-singular (McDonald & Krane, 1977).

### 2.3 Parameter estimation and computation

For a random sample of *n* independent observations, the marginal log-likelihood is: (11)

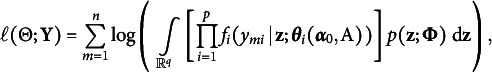

where 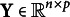 is the observed data matrix with rows given by the *p*-dimensional vectors of observed variables 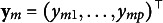 from units 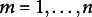 , and 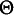 is a *K*-dimensional vector of unknown model parameters. For computational convenience, we parameterize the factor covariance matrix through its Cholesky decomposition 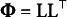 , where 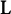 is a lower triangular matrix. When the latent variables are uncorrelated, 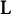 is fixed to the identity matrix, and 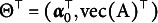 , where “vec” is the vectorization operator that concatenates the free entries of 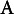 in a vector. In confirmatory settings, where the latent variables are correlated, 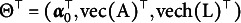 , where “vech” is the half-vectorization operator that concatenates the free lower-triangular entries of 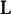 in a vector.

The model parameters are estimated via full-information MML. Let 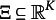 be the reduced parameter space. The maximum likelihood estimate (MLE), denoted by 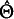 , is: 

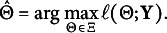



To compute the MLE, we find the solution to the system of (non-linear) score equations 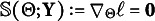 , with entries of the general form (12)

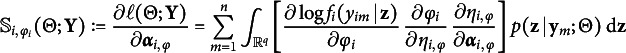

for the vector of factor loadings 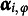 in the measurement equation of the distributional parameter 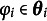 , 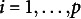 . We provide expressions of the score equations (12) for the GLVM-LSS models introduced in this article in the Section A1 of the Supplementary Material. Scores for the 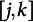 th entry in 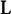 are: (13)

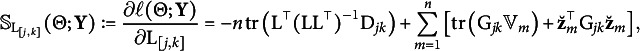

where 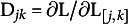 is a square matrix of dimension *q*, with a value of 1 in the 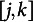 position and zero elsewhere; 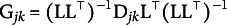 ; and the conditional mean 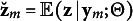 and conditional variance 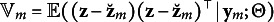 are obtained using the properties of the trace operator and the linearity of the conditional expectation. Details on the computation of 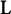 are discussed in the Section A2 of the Supplementary Material.

In most cases, 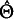 is computed using iterative score-based optimization algorithms. Our estimation strategy begins with a warm-up phase using the Expectation–Maximization (EM) algorithm (Bock & Aitkin, 1981; Dempster et al., 1977), followed by a direct maximization of the marginal log-likelihood with the BFGS quasi-Newton algorithm (Nocedal & Wright, 2006, Chapter 6). The transition between these two algorithms is possible due to the equivalence of the score functions of the complete-data and the marginal log-likelihoods (Louis, 1982).

Upon computation of the MLE, in exploratory settings, an orthogonal or oblique rotation can be applied to the estimated factor loading matrix, 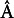 , to obtain a more interpretable and sparse solution (e.g., Jennrich, 2004, 2006; Liu et al., 2023).

Unless the objective function is strictly concave, both the (quasi-)Newton update in the EM algorithm’s M-step and the BFGS algorithm converge to a *local* maximum. This means that the final solution can depend on the initial values chosen. Using different starting values and comparing the resulting marginal log-likelihood estimates is advisable to determine the best solution.

For the one- and two-dimensional models presented in Sections 3 and 4, it is sufficient to evaluate the integrals in the score vector and the information matrices numerically using an ordinary Gauss–Hermite (GH) rule. This can be done with a fixed number of user-defined quadrature points on each dimension of the space of the latent variables. However, the GH approach runs into computational challenges when the dimension of the latent space is high. In such cases, alternatives like adaptive GH quadrature (Rabe-Hesketh et al., 2005; Schilling & Bock, 2005) or stochastic approximation methods (Cai, 2010; Zhang & Chen, 2022) should be considered.

For model selection, we suggest using information criteria for nested (e.g., with restrictions on the model parameters) and non-nested (e.g., GLVM-LSS models with different distributions on the measurement model) models. The Akaike Information Criterion (AIC, Akaike, 1974) and the Bayesian Information Criterion (BIC, Schwarz, 1978) are popular criteria to evaluate model fit. The AIC and BIC often concur and are commonly used together in applied research (Kuha, 2004). However, in case of divergent results, we suggest referring to the AIC when dimensionality reduction is the primary goal, as it favors (expected) predictive performance (Shao, 1997); while using the BIC when the study involves substantive interpretation of the latent variables, as it favors consistent model selection (Nishii, 1984).

## 3 Empirical applications

We present two empirical applications that follow from the GLVM-LSS examples introduced in Section 2.1.

### 3.1 ANES 2020: “Thermometer” variables

The ANES 2020 dataset contains “feeling thermometer” variables on social groups, including sexual orientation and gender identity groups (Gay men and Lesbians, Transgender people), social and political movements (Feminists, #MeToo and BLM movements), and groups that were trending in the news during 2020 (labor unions, journalists, scientists) from the post-election sample of the 2020 American National Election Study (ANES)^2^. Participants rate their feelings toward social groups on a scale of 0–100, where higher ratings indicate more favorable attitudes.

The ANES thermometer variables have been used in some studies as a substitute for political orientation and measures of personal and societal values (e.g., Abelson et al., 1982; Guth, 2019; Krasa & Polborn, 2014). Similarly, they provide insights into an individual’s position on a *conservative-progressive* belief scale. We present results for the one-factor Beta model^3^ .

We model the observed variables using the heteroscedastic Beta factor model discussed in Section 2.1, i.e., 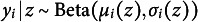 , 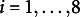 . We scale the responses by 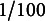 so the observed variables are within the interval 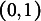 . We also replace extreme responses on the boundaries of the interval with numerical values that are arbitrarily close to 0 and 1 ( 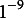 and 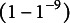 , respectively). We exclude individuals with incomplete interviews or technical errors in their answers from the analysis, treat responses of *“Don’t know”*, *“Don’t recognize”*, and *“Refuse”* as missing data. The resulting sample consists of 7253 respondents.

**Figure 1:**
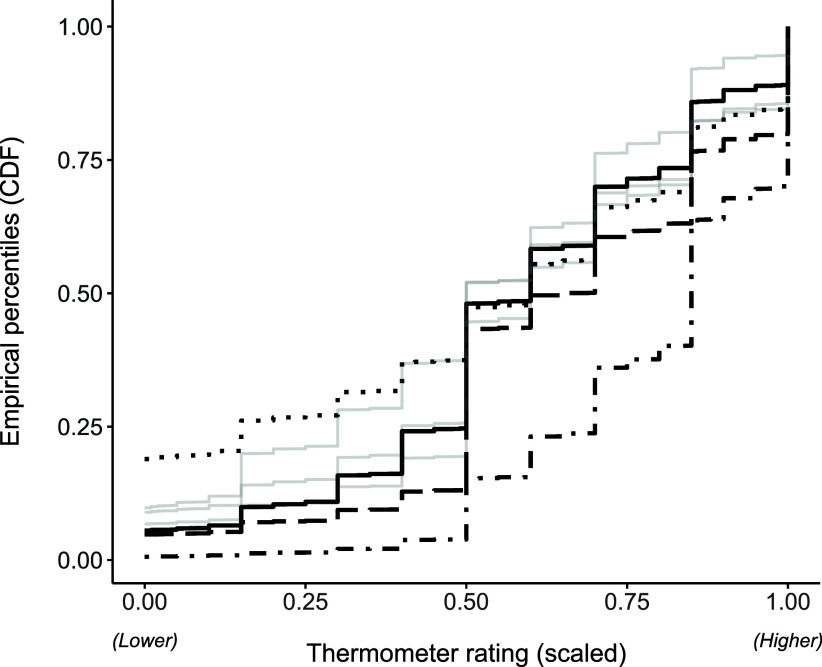
ANES 2020: Empirical cumulative distribution function (ECDF). *Note*: Highlighted variables: *Feminists* (solid line, 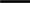), *Gay men and Lesbians* (dashed line, 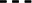), *BLM movement* (dotted line, 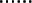), and *Scientists* (dash-dot line, 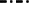).

The Section A4a of the Supplementary Material presents descriptive statistics for the observed variables. Most variables have negatively skewed marginal empirical distributions and negative excess kurtosis, except item *Scientists*. The empirical cumulative distribution functions (ECDF) for the observed variables are displayed in Figure 1. Although the thermometer ratings are measured on a continuous scale, respondents tend to round their answers to the nearest 5 or 10, resulting in a stepped appearance in the ECDFs. Some questions show a higher frequency of extreme responses (either zero or one). Most responses tend to cluster around 0.5, suggesting that respondents are reluctant to take a clear position on issues related to the conservative-progressive belief spectrum. We estimated a baseline (homoscedastic) model, which assumes a constant scale parameter, and an alternative (heteroscedastic) model, which allows the scale parameter to vary based on the latent variable. The heteroscedastic model was selected using the AIC and BIC criteria, as detailed in Table 1. Additionally, Table 2 presents the parameter estimates along with their corresponding estimated (SEs) for the heteroscedastic model.

**Table 1:** ANES 2020: AIC and BIC for the homoscedastic and heteroscedastic Beta factor models. Information criteria for the best fitting model are in bold.

Model	AIC	BIC	*K*
Beta ( 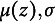 )	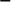 270,078.20	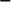 269,912.86	24
Beta ( 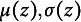 )	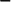 **271,002.07**	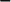 **270,781.62**	32

*Note*: 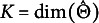 is the number of parameters in the corresponding model.

**Table 2:** ANES 2020: Estimated (Est.) coefficients and their standard errors (SE) for the heteroscedastic Beta factor model

	Location parameter 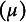 measurement equation				Scale parameter 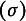 measurement equation			
Item	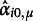		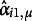		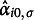		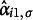	
	Est.	SE	Est.	SE	Est.	SE	Est.	SE
Gay men and Lesbians	1.00	(0.03)	1.54	(0.02)	0.52	(0.01)	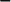 0.25	(0.01)
Transgender people	0.61	(0.03)	1.70	(0.02)	0.36	(0.01)	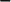 0.15	(0.01)
Feminists	0.49	(0.02)	1.56	(0.02)	0.29	(0.01)	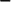 0.14	(0.01)
#MeToo movement	0.47	(0.03)	1.65	(0.03)	0.57	(0.01)	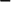 0.28	(0.01)
BLM movement	0.12	(0.02)	1.32	(0.03)	1.28	(0.01)	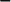 0.25	(0.01)
Labor Unions	0.46	(0.02)	0.83	(0.01)	0.77	(0.01)	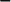 0.16	(0.01)
Journalists	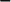 0.06	(0.02)	1.21	(0.02)	0.71	(0.01)	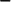 0.16	(0.01)
Scientists	1.72	(0.02)	0.84	(0.02)	0.71	(0.01)	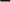 0.19	(0.01)

The initial values for the estimation algorithm were chosen by conducting a principal component analysis on the observed data matrix. We then used the first principal component as the explanatory variable in a series of independent distributional regression analyses with a Beta distribution for the outcome variable. To explore potential local solutions, we tested various random starting values; however, the results remained consistent with those reported below.

We first discuss the results of the location parameter equations. The estimated slopes ( 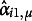 ’s) are positive and statistically significant, indicating that more progressive individuals tend to rate these groups higher on average. In addition, lower intercept values ( 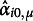 ’s) indicate that the items are perceived as more challenging, meaning a more progressive position on the latent scale is necessary to achieve at least 50% on these items.

In discussing the scale parameter equations, all the estimated slopes ( 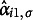 ’s) are negative and statistically significant. This indicates that individuals on the “progressive” side of the latent scale tend to hold more homogeneous views about these groups than those on the ‘conservative’ side, in line with previous findings in public opinion research literature. The estimated intercepts ( 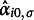 ’s) determine the conditional variance for the “average” position on the latent scale (i.e., 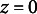 ). Larger intercepts imply greater heterogeneity in people’s responses near the middle point of the latent scale.

**Figure 2:**
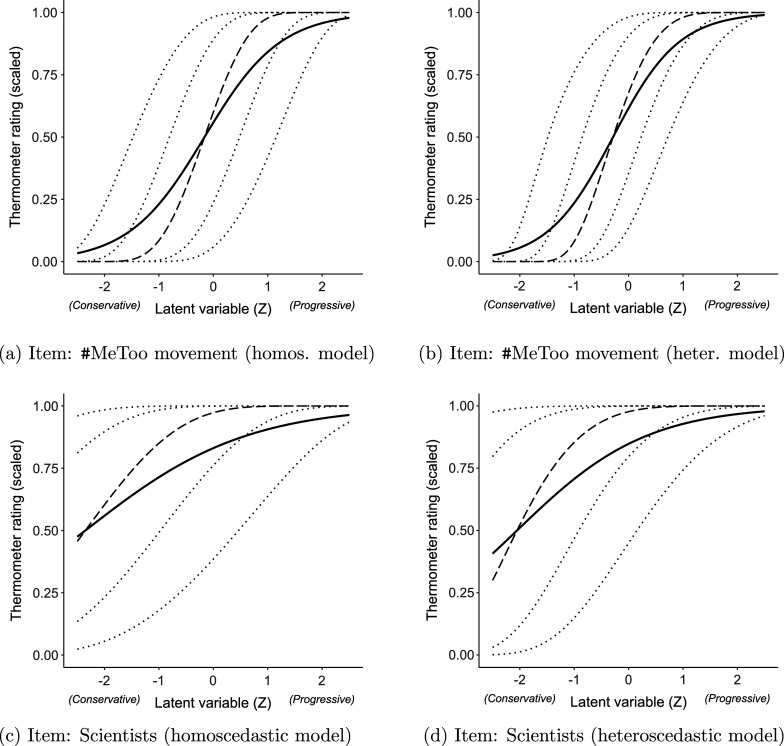
ANES 2020: Fitted conditional expected values (solid line, ——), median (dashed line, ---), and percentiles (dotted lines, 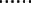).

A comparison of the fitted (conditional) distribution implied by the homoscedastic and heteroscedastic models for selected variables is shown in Figure 2. The plot includes the fitted mean, median, and percentiles (10th, 25th, 75th, and 90th) for both models. The homoscedastic model (shown in Figure 2a and 2c) does not effectively capture the asymmetries in the conditional distributions of the observed variables along the latent scale. In contrast, the heteroscedastic model, illustrated in Figure 2b and 2d, successfully captures these asymmetries.

### 3.2 PISA 2018: A joint model for IRs and RTs

The second dataset was obtained from the 2018 PISA computer-based mathematics exam. We focused on a sample of Brazilian students who answered nine binary items from the first testing booklet. Only individuals who provided complete responses were included, resulting in a final sample size of 1,280 students. RTs for each binary item were recorded in logarithmic minutes. Descriptive statistics for the IRs and log RTs are available in the Section A4b of the Supplementary Material.

RTs provide valuable information about a student’s ability and test-taking strategies and are also helpful in item calibration and test design (van der Linden, 2007, 2008; van der Linden & Guo, 2008; van der Linden et al., 2010). A hierarchical model for speed and accuracy on test items was initially proposed in van der Linden (2007, 2009), and later extended in Bolsinova & Molenaar (2018); Bolsinova et al. (2017); Molenaar et al. (2015) and others. For a review of models involving items and RTs, see De Boeck & Jeon (2019).

The baseline model in van der Linden (2007) assumes that the log-RTs follow a Normal distribution, with only the conditional mean (location parameter) depending on the latent speed factor. Factor loadings for log-RTs are fixed^4^, but the variance of the latent speed factor is freely estimated. We extend this model by employing the SN distribution, which models varying heterogeneity and skewness in the log-RTs along the latent speed factor as described in Section 2.1. Variances for the latent ability and latent speed factors are fixed at 1, while the factor loadings are freely estimated. Higher-order moments of RTs can provide valuable insight into students’ test-taking strategies, thought processing during high-stakes standardized tests, and information on item quality.

**Figure 3:**
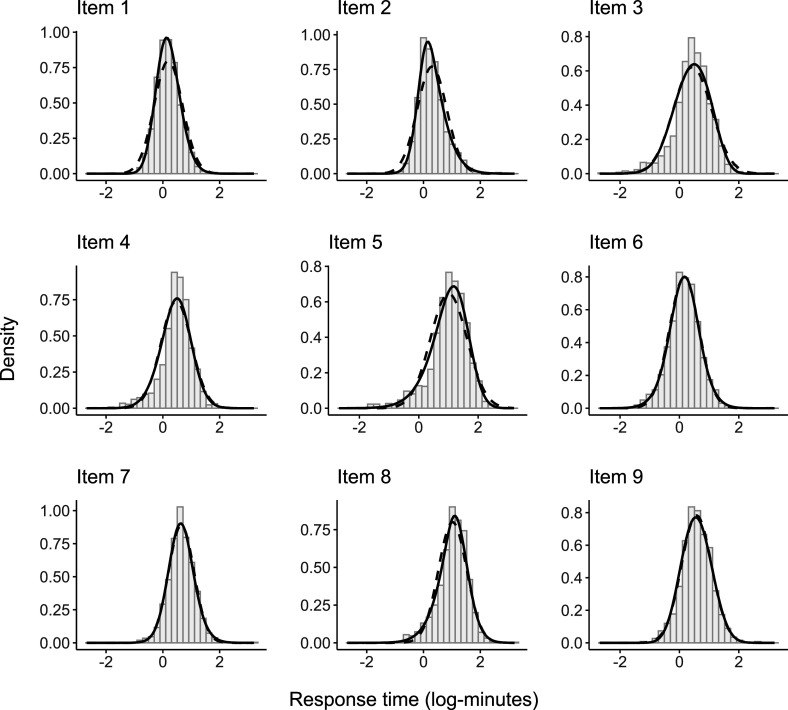
PISA 2018: Empirical and model-implied marginal distributions for response times (in log-minutes). *Note*: The solid line (——) is the SN model, and the dashed line (---) the Normal model.

The empirical and model-implied marginal distributions of the RTs in log minutes are displayed in Figure 3. We observed that the majority of the log-RTs showed some degree of skewness. The model fit improved when the log-RTs were assumed to follow an SN distribution (solid line) rather than a Normal distribution (dashed line).

We estimated seven increasingly complex models, including the proposed confirmatory factor model discussed in Section 2.1, using the full-information maximum likelihood procedure described in Section 2.3. We tried different starting values to check for local solutions, and the results remained consistent across estimations. We began the estimation process by implementing a warm-start strategy, which involved a PCA decomposition on the matrix of observed variables. We then retained 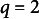 principal components to use them as observed covariates in a series of distributional regressions with IRs and log-RTs as outcomes. The two-dimensional integrals were numerically evaluated using the GH quadrature, with 45 quadrature points in each latent dimension (a total of 2025 quadrature points).

Models 1 to 3 assume the log-RTs follow a conditional Normal distribution. Model 1 serves as the baseline hierarchical model described in van der Linden (2007). Model 2 allows to freely estimate the factor loadings in the log-RT model, similar to the ‘unrestricted model’ in Molenaar et al. (2015), while fixing the variance of the latent speed factor to 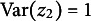 for identification purposes. Model 3 is a heteroscedastic version of Model 2. In models 4 to 7, we assume that the log-RTs follow a conditional SN distribution. In Model 4, only the location parameter ( 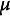 ) depends on the latent speed factor, while the scale ( 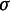 ) and shape ( 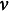 ) parameters remain constant. Models 5 and 6 model 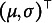 and 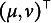 as functions of 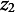 , respectively. Model 7, the full-SN model, treats all distributional parameters as functions of the latent speed trait. In all cases, the IRT model for the IRs remains consistent with what was described above, with the equation given in (6). Results are presented in Table 3. Notably, models with SN log-RTs demonstrate better model fit compared to those with Normal log-RTs. Model 7 provides the best fit based on its AIC and BIC values.

**Table 3:** PISA 2018: AIC and BIC for GLVM-LSS for the joint modeling of item responses and response times. Information criteria for the best fitting model are in bold.

Model	AIC	BIC	*K*
1. Bernoulli ( 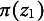 ) + Normal ( 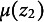 , fixed 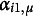 ’s)	26,173.61	26,369.49	38
2. Bernoulli ( 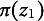 ) + Normal ( 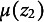 )	25,908.68	26,145.79	46
3. Bernoulli ( 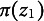 ) + Normal ( 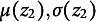 )	25,754.91	26,038.42	55
4. Bernoulli ( 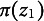 ) + Skew-Normal ( 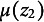 )	25,326.08	25,609.58	55
5. Bernoulli ( 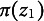 ) + Skew-Normal ( 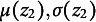 )	25,281.44	25,611.33	64
6. Bernoulli ( 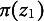 ) + Skew-Normal ( 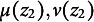 )	25,232.76	25,562.66	64
7. Bernoulli ( 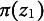 ) + Skew-Normal ( 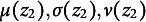 )	**25,172.12**	**25,548.41**	73

*Note*: 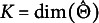 is the number of parameters in the corresponding model.

Estimates for the intercepts, loadings, and factor correlation in Model 7, along with their corresponding estimated SEs, are presented in Table 4. The interpretation of the intercepts and slopes in the equations for the location parameter of the IRs ( 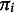 ’s) and log-RTs ( 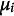 ’s) is straightforward. The 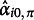 ’s and 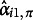 ’s represent the difficulty and discrimination parameters for the IRs. In other words, items with lower 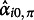 values are considered more difficult, while those with higher 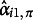 values are seen as having more discrimination power.

**Table 4:** PISA 2018: Estimated coefficients (Est.) and Standard Errors (SE) for a joint model of item responses and response times (Model 7)

	Estimated coefficients in the equation for item responses (IR)				Estimated coefficients in the equations for (log-)response times (log-RT)											
	Location parameter 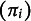				Location parameter 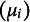				Scale parameter 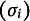				Shape parameter 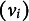			
Item	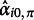		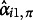		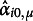		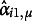		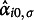		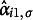		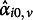		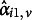	
	Est.	SE	Est.	SE	Est.	SE	Est.	SE	Est.	SE	Est.	SE	Est.	SE	Est.	SE
Item 1	0.64	(0.07)	0.79	(0.10)	0.19	(0.01)	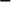 0.17	(0.01)	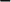 0.95	(0.02)	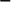 0.03	(0.02)	0.63	(0.14)	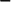 0.03	(0.16)
Item 2	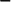 0.47	(0.07)	1.03	(0.11)	0.30	(0.01)	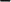 0.24	(0.01)	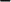 0.89	(0.02)	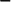 0.10	(0.02)	1.56	(0.16)	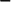 0.78	(0.19)
Item 3	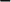 0.04	(0.10)	1.94	(0.21)	0.42	(0.02)	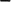 0.25	(0.02)	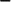 0.61	(0.02)	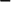 0.08	(0.02)	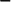 1.07	(0.14)	0.81	(0.16)
Item 4	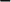 0.69	(0.08)	0.96	(0.11)	0.45	(0.02)	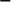 0.34	(0.01)	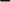 0.87	(0.02)	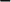 0.05	(0.02)	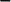 1.45	(0.14)	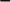 0.33	(0.16)
Item 5	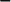 2.85	(0.23)	2.28	(0.25)	1.00	(0.02)	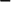 0.36	(0.02)	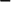 0.68	(0.02)	0.14	(0.02)	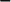 1.04	(0.14)	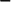 0.32	(0.21)
Item 6	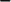 0.91	(0.07)	0.32	(0.08)	0.16	(0.02)	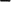 0.36	(0.01)	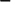 0.97	(0.02)	0.03	(0.03)	0.11	(0.13)	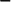 0.88	(0.22)
Item 7	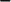 4.84	(0.45)	2.52	(0.34)	0.65	(0.01)	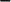 0.33	(0.01)	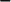 1.15	(0.02)	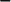 0.04	(0.02)	0.35	(0.15)	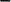 0.58	(0.18)
Item 8	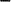 3.64	(0.32)	2.36	(0.29)	1.02	(0.02)	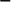 0.39	(0.01)	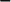 1.02	(0.02)	0.12	(0.03)	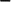 1.22	(0.22)	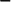 1.61	(0.30)
Item 9	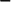 2.73	(0.17)	1.45	(0.16)	0.58	(0.02)	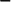 0.30	(0.01)	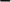 0.90	(0.02)	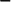 0.00	(0.02)	0.30	(0.10)	0.36	(0.14)
Estimated latent correlation ( 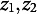 ): 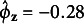 (SE: 0.04)																

The 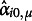 ’s in log-RTs represent the average log-RTs for 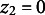 , also known as the item’s average time intensity (van der Linden, 2007). The estimated slopes 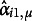 in the equation for the location parameter of the log-RTs are all negative. This suggests that individuals with a higher latent speed trait will respond faster to any given item.

The estimated correlation between the latent ability and the speed factor is 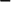 0.28 (SE 0.04), suggesting that test takers with higher latent ability generally take longer to respond. This result aligns with previous studies on the speed-accuracy trade-off, which indicates that individuals who respond slowly make fewer mistakes compared to those who respond quickly and make more mistakes (e.g., van der Linden, 2007, and Heitz, 2014 for a general overview on the subject). Previous studies have found correlations between the latent ability and the latent speed trait of similar sign and magnitude in large-scale educational testing of quantitative subjects (e.g., van der Linden & Guo, 2008).

**Figure 4:**
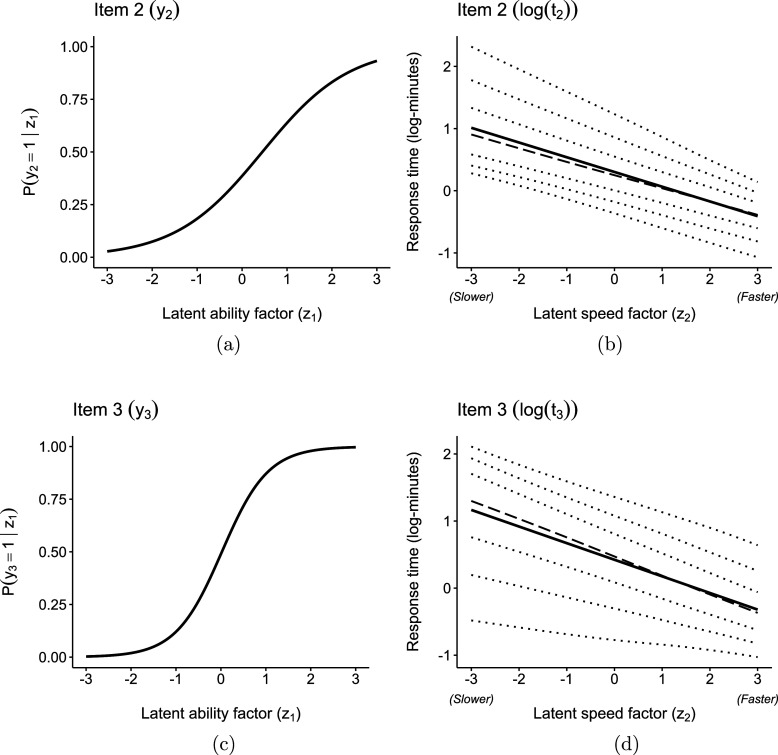
PISA 2018: Fitted conditional expected values (solid line, ——), median (dashed line, ---), and percentiles (dotted lines, 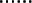) for IR and log-RT for items 2 and 3.

**Figure 5:**
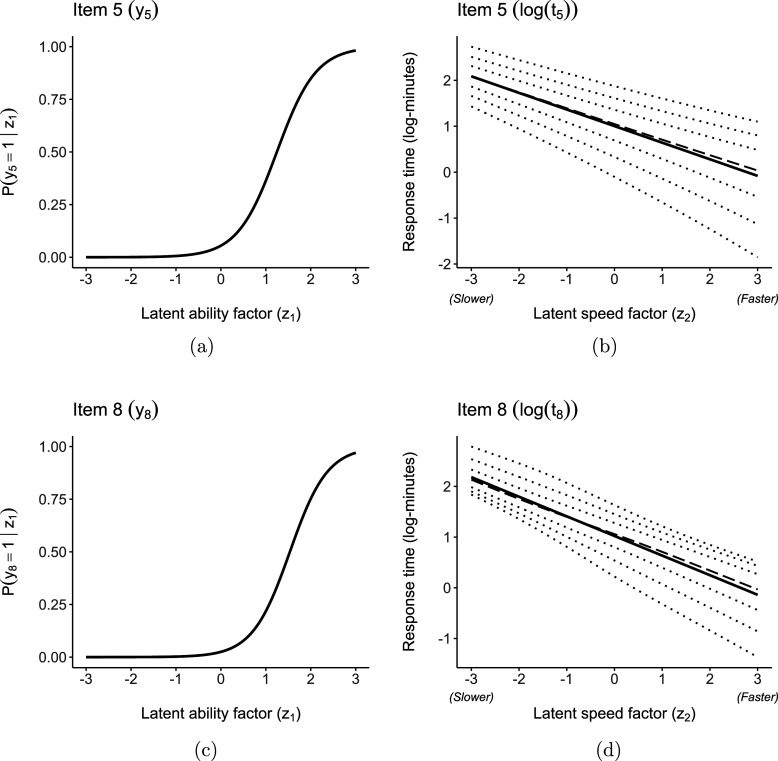
PISA 2018: Fitted conditional expected values (solid line, ——), median (dashed line, ---), and percentiles (dotted lines, 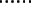) for IR and log-RT for items 5 and 8.

The equations for the scale (standard deviation) and shape (skewness) parameters of the log-RTs provide valuable information about the items and RTs. The estimates 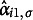 and 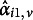 indicate that some log-RTs exhibit heteroscedasticity (items 2, 3, 4, 5, 8) and varying skewness (items 2, 3, 4, 6, 7, 8, 9) in their log-RTs as the latent speed factor changes. Selected item characteristic curves (ICC) for the IRs and the fitted SN conditional distributions for the log-RTs (parameterized by the coefficients in Table 4) are shown in Figures 4 and 5. The (conditional) mean, median, and percentiles (0.025, 0.10, 0.25, 0.75, 0.90, and 0.975) for the log-RTs are plotted to illustrate how the distribution’s shape changes as the latent speed factor dimension varies.

Figures 4a and 4b illustrate item 2, while Figure 4c and 4d depict item 3. The figures show how the variance of 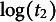 and 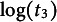 changes as we move along the latent speed factor dimension—the conditional skewness, however, changes in opposite directions. For instance, 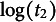 is positively skewed for individuals in the left tail of the latent speed factor, while 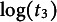 is negatively skewed for the same group of students. On the other hand, the RTs’ distributions are symmetric for individuals on the right tail of the speed factor dimension. Figure 5a and 5b depict item 5, while Figure 5c and 5d correspond to item 8. The estimated positive slope for the scale parameter suggests a larger variance in the RTs for individuals on the upper tail of the latent speed factor distribution. These items are among the most difficult ones, with higher 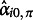 values, and also require more time on average, with higher 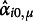 values. Furthermore, they also exhibit varying skewness parameters. For example, for item 8, the direction of the skewness changes depending on the location along the latent speed factor scale. These results might suggest differences in item characteristics, such as the wording, task, difficulty, or cognitive processes required for their completion.

## 4 Simulation studies

We performed several simulation studies to evaluate the accuracy of the parameter estimates obtained through the MML algorithm explained in Section 2.3, along with their corresponding SEs. Simulations were conducted in R (R Core Team, 2022) using the package glvmlss, with underlying functions programmed in C++ using packages Rcpp (Eddelbuettel, Francois, Allaire, et al., 2023), RcppArmadillo, (Eddelbuettel, Francois, Bates, et al., 2023), and RcppEnsmallen (Balamuta & Eddelbuettel, 2018). Code and replication files are available at https://github.com/ccardehu/glvmlss.

### 4.1 Simulation study I

The first simulation study closely resembles the empirical application discussed in Section 3.2. In this study, we examine observed continuous variables within the interval 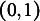 , which are assumed to follow a location-scale parametrization of the Beta distribution. Specifically, the heteroscedastic Beta factor model features location and scale parameters that are defined as functions of a single latent variable, i.e., 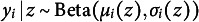 .

The values for the population parameters in the location parameter equation are selected from two uniform distributions: 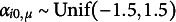 and 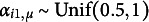 . The signs of the 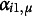 ’s are assigned randomly with a probability of 0.5. The parameters for the scale equation are sampled from the uniform distribution 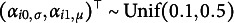 , with the signs of the slopes also assigned randomly. We generate the true parameters in this way to ensure that the conditional densities 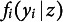 are uni-modal. Although the Beta distribution allows for bimodal densities for certain combinations of 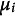 and 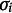 , this is not common in the applications of interest (see Noel (2014) for a unidimensional unfolding Beta factor model that handles the bi-modality of the observed variables). The integrals involved in parameter computation were numerically evaluated using a fixed-point Gauss–Hermite rule with 100 quadrature points.

We generated 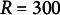 datasets for each of the 12 conditions. These conditions were created by combining four different sample sizes (200, 500, 1,000, and 5,000) with three different numbers of observed variables (5, 10, and 20). The quality of the estimated parameters was assessed by the mean squared error (MSE): 

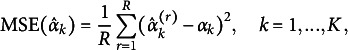

and the absolute bias (AB): 

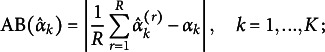

where 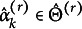 is an arbitrary parameter estimate from the *r*^th^ replication, and 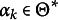 is the true value for the model parameter. We report the average MSE (AvMSE) and the average AB (AvAB) separately for the intercepts and slopes in the equations of the location ( 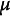 ) and scale ( 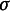 ) parameters indexing the Beta distribution. For completeness, we include box-plots with the simulation results for individual parameters ( 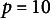 ) in the Section A5 of the Supplementary Material. Similar results hold for other numbers of observed variables.

To evaluate the accuracy of the estimated SEs and corresponding confidence intervals, we calculate the average coverage rate across replications for various intercepts and slopes in the location and scale parameters equations. The coverage rate (CR) of the 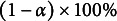 confidence interval for a parameter estimate 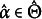 is: 

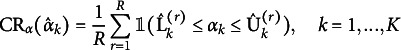

where 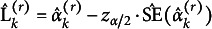 is the sample-dependent lower bound, 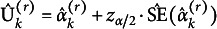 is the sample-dependent upper bound, and 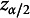 corresponds to the 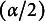 th quantile of the standard Normal distribution. The customary level of the nominal rate is 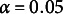 . Coverage rates close to 0.95 indicate a good estimation of the 95% confidence intervals. We report the average CR (AvCR) for intercepts and slopes separately.

Table 5 gives all the results. As expected, the AvMSE and the AvAB tend to decrease as the sample size increases for all values of *p*. As for the AvCRs, they reach the nominal level as the sample size increases. It is worth noting that estimated SEs are slightly overestimated for smaller sample sizes, leading to more conservative confidence intervals.

**Table 5:** Simulation Study I: Average Mean Squared Error (AvMSE), Average Absolute Bias (AvAB), Average Coverage Rate (AvCR), and Average computation time in minutes (CT) for the MLE of an LVM with Beta distributed observed variables, by test length and sample size

		Average MSE (AvMSE)				Average AB (AvAB)				Average CR (AvCR)				
		Inter.	Load.	Inter.	Load.	Inter.	Load.	Inter.	Load.	Inter.	Load.	Inter.	Load.	Average
*p*	*n*	( 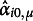 )	( 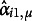 )	( 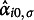 )	( 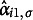 )	( 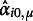 )	( 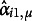 )	( 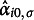 )	( 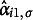 )	( 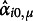 )	( 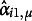 )	( 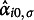 )	( 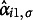 )	CT (mins.)
	200	0.0105	0.0146	0.0102	0.0081	0.0037	0.0058	0.0240	0.0058	0.9587	0.9720	0.9587	0.9720	0.0415
	500	0.0045	0.0055	0.0039	0.0031	0.0021	0.0050	0.0081	0.0058	0.9507	0.9633	0.9547	0.9660	0.1194
	1,000	0.0021	0.0026	0.0018	0.0015	0.0016	0.0050	0.0038	0.0029	0.9553	0.9573	0.9540	0.9620	0.2286
5	5,000	0.0004	0.0006	0.0004	0.0003	0.0008	0.0044	0.0020	0.0032	0.9580	0.9420	0.9527	0.9473	1.4237
	200	0.0117	0.0109	0.0079	0.0064	0.0050	0.0066	0.0153	0.0042	0.9630	0.9810	0.9640	0.9917	0.0851
	500	0.0041	0.0042	0.0029	0.0026	0.0028	0.0060	0.0073	0.0036	0.9663	0.9733	0.9583	0.9723	0.2409
	1,000	0.0021	0.0021	0.0014	0.0014	0.0012	0.0042	0.0040	0.0042	0.9570	0.9600	0.9590	0.9577	0.4608
10	5,000	0.0004	0.0004	0.0003	0.0003	0.0009	0.0038	0.0014	0.0037	0.9517	0.9470	0.9520	0.9403	3.0741
	200	0.0103	0.0094	0.0067	0.0059	0.0060	0.0045	0.0161	0.0050	0.9860	0.9973	0.9853	0.9967	0.1842
	500	0.0042	0.0037	0.0026	0.0024	0.0034	0.0050	0.0056	0.0039	0.9670	0.9790	0.9653	0.9780	0.4937
	1,000	0.0020	0.0018	0.0013	0.0012	0.0020	0.0039	0.0040	0.0030	0.9605	0.9707	0.9555	0.9693	1.0251
20	5,000	0.0004	0.0004	0.0003	0.0003	0.0012	0.0031	0.0010	0.0028	0.9503	0.9452	0.9477	0.9440	6.5482

*Note*: Performance measures are computed for the estimated parameters 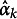 in the location loading matrix ( 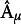 ) and scale loading matrix ( 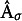 ).

### 4.2 Simulation study II

The second study resembles the empirical application in Section 3.2. We study the finite sample performance of a confirmatory GLVM-LSS model with two latent variables ( 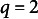 ) and sixteen observed variables ( 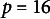 ). The first eight variables are distributed as Bernoulli, conditional on the first factor, 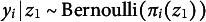 for 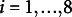 . The remaining eight variables are distributed SN, conditional on the second factor, 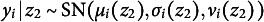 for 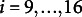 . The latent variables follow a multivariate standard Normal distribution, 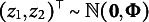 , where 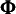 is a correlation matrix with non-zero off-diagonal entries denoted by 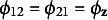 .

The simulation study considers three sample sizes, 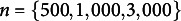 . The intercepts, slopes, and factor correlation are fixed to the parameter estimates for items 1–7 and 9 in Table 4 ^5^. As before, we compute the AvMSE and AvAB for the estimated parameters and assess the properties of the estimated confidence intervals via the AvCR. The above measures were obtained from 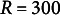 independently simulated datasets. The multidimensional integrals were numerically evaluated using the Gauss–Hermite rule with 35 quadrature points on each latent dimension (a total of 1,225 quadrature points).

For better numerical stability, we estimate the model parameters by letting the EM algorithm run for a large number of iterations, using a gradient descent update rule with adaptive learning rate^6^. After 500 iterations, the algorithm switches to the quasi-Newton direct maximization step. Table 6 presents the results for each sample size.

**Table 6:** Simulation Study II: Average Mean Squared Error (AvMSE), Average Absolute Bias (AvAB), Average Coverage Rate (AvCR), and Average computation time in minutes (CT) for the MLE of a confirmatory GLVM-LSS with Bernoulli and Skew-Normal distributed observed variables, by sample size and type of parameter

		Average MSE	Average AB	Average CR	Average CT
*n*	Parameter	(AvMSE)	(AvAB)	(AvCR)	(AvCT, mins.)
	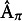	0.1166	0.0398	0.9631	24.996
	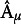	0.0006	0.0012	0.9613	
	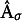	0.0017	0.0039	0.9672	
	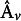	0.2033	0.0498	0.9775	
500	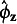	0.0036	0.0069	0.9860	
	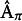	0.0553	0.0173	0.9599	70.152
	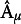	0.0003	0.0009	0.9597	
	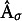	0.0008	0.0023	0.9607	
	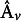	0.0775	0.0212	0.9626	
1,000	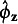	0.0017	0.0013	0.9730	
	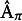	0.0203	0.0100	0.9496	197.136
	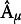	0.0001	0.0003	0.9575	
	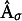	0.0003	0.0010	0.9563	
	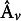	0.0209	0.0101	0.9581	
3,000	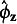	0.0005	0.0006	0.9867	

*Note*: Performance measures are computed for the estimated parameters in the loading matrix for the Bernoulli items ( 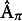 ); the loading matrices for the location ( 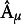 ), scale ( 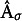 ), and shape ( 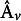 ) parameters for the Skew-Normal items; and the correlation between the latent variables ( 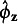 ).

For simplicity, we present the aggregate results for intercepts and factor loadings in each matrix 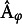 , 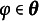 . In all cases, the AvMSE and AvAB decrease with sample size, as expected. The results in Table 6 also suggest that the factor correlation 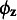 is consistently estimated. Coverage rates are around nominal levels for medium and large samples, while, as discussed previously, the confidence intervals for the smaller sample size are slightly conservative.

## 5 Discussion

This article presents a general framework for latent variable modeling called GLVM-LSS. In this framework, all the distributional parameters characterizing each observed variable’s conditional distribution are modeled as functions of linear combinations of the latent variables. In this respect, the (conditional) mean and higher-order (conditional) moments of the observed variables—expressed in terms of the corresponding location, scale, and shape parameters—are also considered functions of the latent variables. The GLVM-LSS offers a wide range of possibilities for modeling complex multivariate datasets by allowing the modeling of data displaying heteroscedasticity, excess skewness, excess kurtosis, zero/one/maximum value inflation, heaping, truncation, or censoring. Model parameters are estimated via full-information maximum likelihood. We demonstrate the effectiveness of our framework by presenting two GLVM-LSS applications using real-world data in public opinion research and educational testing. Our proposed method is implemented in the R package glvmlss, available online at https://github.com/ccardehu/glvmlss.

The GLVM-LSS framework has numerous potential applications in empirical research areas where modeling higher-order moments of observed variables as functions of latent variables is relevant. For instance, in ecological momentary assessment designs, researchers are interested in individuals’ emotional response variability, in addition to the deviations from their baseline mood (Hedeker et al., 2006, 2008, 2012; Wang et al., 2012). While these models include random effects, incorporating a latent variable specification could enrich the analysis. Another example is item quality control in educational testing (Hessen & Dolan, 2009). Heteroscedastic items often exhibit low discrimination power, which can reduce test accuracy if not revised or removed. From a dimensionality reduction perspective, it is desirable to preserve as much information as possible and to model the essential aspects of the observed data. Modeling the entire (conditional) distribution can result in better data recovery than simply modeling the (conditional) mean (Shen & Meinshausen, 2024).

While the GLVM-LSS is a flexible tool for modeling multivariate data with latent variables, there are still opportunities for improvement in future research. Currently, the model’s implementation in the glvmlss package computes numerical integrals in the MML estimation algorithm and factor scoring procedure using a fixed-point Gaussian–Hermite quadrature rule. This approach limits the number of latent variables that can be included in the model without encountering computational bottlenecks. Future updates of the glvmlss package will aim to address this limitation by incorporating either adaptive Gaussian–Hermite quadrature rules (Rabe-Hesketh et al., 2005) or stochastic approximation methods (Cai, 2010; Zhang & Chen, 2022). These alternatives for numerical integration have been shown to produce fast and accurate solutions and thus should be explored further in the context of the GLVM-LSS.

As discussed earlier, one potential extension of the GLVM-LSS framework is to incorporate observed covariates into both the measurement and structural equations. This addition could enhance the framework’s usefulness in latent regression contexts and aid in testing for measurement invariance or DIF. Also, many datasets in the social sciences suffer from non-ignorable missingness, and thus, one can extend the GLVM-LSS and implement methods developed, for example, in O’Muircheartaigh & Moustaki (1999).

Moreover, increasing the flexibility to model distributional parameters also raises the number of parameters that need to be estimated, which can lead to computational and interpretational challenges. A regularized estimation approach for the GLVM-LSS can be developed to mitigate these issues. This method aims to produce more sparse and interpretable factor loading solutions while also facilitating model selection through the appropriate choice of the regularization parameter (e.g., Cárdenas-Hurtado, 2023; Geminiani et al., 2021).

Another extension of the GLVM-LSS framework involves relaxing the linearity assumption in the specification of the (linear) predictor 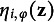 . Following the generalized additive model specification in the GAMLSS regression framework, for each distributional parameter 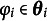 we could model the relationship between the observed and latent variables using splines (de Boor, 2001; Ramsay & Abrahamowicz, 1989). This approach extends previous research in non-linear LVMs using polynomials (McDonald, 1962, 1967; Rizopoulos & Moustaki, 2008; Yalcin & Amemiya, 2001). It also has connections with existing models in the LVM literature, such as the unidimensional semi-parametric IRT models (e.g., Falk & Cai, 2016a,b; Johnson, 2007; Ramsay & Winsberg, 1991; Rossi et al., 2002).

## Supporting information

Cárdenas-Hurtado et al. supplementary material Cárdenas-Hurtado et al. supplementary material

## Data Availability

The associated glvmlss package and replication files are available online at https://github.com/ccardehu/glvmlss.

## Footnotes

1 For modeling convenience, the shape parameter is restricted to the  interval. We achieve this by applying a monotonic transformation of the shape parameter in the “centered” parametrization outlined in Azzalini (1985) and described in Azzalini & Capitanio (1999). We refer the readers to Section A1 of the Supplementary Material for further details.

2 American National Election Studies, 2021. (www.electionstudies.org). Full Release (dataset and documentation). July 19, 2021 version. These materials are based on work supported by the National Science Foundation under grant numbers SES-1444721, 2014-2017, the University of Michigan, and Stanford University.

3 We also explored two-factor Beta models, but careful analysis of the expected information matrix (evaluated at the MLE) reveals that the heteroscedastic Beta factor model with  is not of full rank. Thus, the model is not empirically identified for this dataset.

4 Slopes in the log-RTs equations are implicitly fixed to  1 in van der Linden’s hierarchical model.

5 These eight items were chosen randomly from the nine in the original PISA 2018 application.

6 The initial learning rate was  , and it was halved if the objective function in the EM algorithm did not increase between iterations.

## Supplementary material

The supplementary material for this article can be found at https://doi.org/10.1017/psy.2025.7.
