# Supplementary material for: Generalized Latent Variable Models for Location, Scale, and Shape parameters
Source: Psychometrika. 2025 Mar 6;90(3):932–56. doi: 10.1017/psy.2025.7 (PMC12483711; doi:10.1017/psy.2025.7)
Supplement: Cárdenas-Hurtado et al. supplementary material [file S0033312325000079sup001.pdf]

Online Supplementary Material for: 'Generalized Latent Variable Models for Location, Scale, and Shape parameters'

Camilo A. Cárdenas-Hurtado* Irini Moustaki†

Yunxiao Chen‡ Giampiero Marra§

A1. Score Vectors

Recall the general expression in equation (11) for the entries in the score vector corresponding to the factor loadings:

$$\mathbb{S}_{i,\varphi_i}(\Theta;\mathbf{Y}) = \sum_{m=1}^{n} \int_{\mathbb{R}^q} \left[\frac{\partial \log f_i(y_{im}\,|\,\mathbf{z})}{\partial \varphi_i} \, \frac{\partial \varphi_i}{\partial \eta_{i,\varphi}} \, \frac{\partial \eta_{i,\varphi}}{\partial \boldsymbol{\alpha}_{i,\varphi}} \right] p(\mathbf{z}\,|\,\mathbf{y}_m;\Theta)\, \mathrm{d}\mathbf{z} \tag{A1.1}$$

Below we give expressions for the bracketed arguments in (A1.1) for the heteroscedastic Beta factor model and for the Skew-Normal distributed log-response times in the joint model for item responses in response times in Section 2.1.

Heteroscedastic Beta Factor Model

We first discuss the standard parametrization of the Beta distribution. Conditional on the latent variables $\mathbf{z}$, let $y_i\,|\,\mathbf{z} \sim \text{Beta}_0(\alpha_i(\mathbf{z}), \beta_i(\mathbf{z}))$ be an observed variable following a Beta

*Corresponding author. Department of Statistics, LSE, c.a.cardenas-hurtado@lse.ac.uk

†Department of Statistics, LSE, i.moustaki@lse.ac.uk

‡Department of Statistics, LSE, y.chen186@lse.ac.uk

§Department of Statistical Science, UCL, giampiero.marra@ucl.ac.uk

distribution with parameters $\alpha_i(\mathbf{z}) > 0$ and $\beta_i(\mathbf{z}) > 0$, for $i = 1, \ldots, p$. The log-density is given by

$$\begin{aligned}\log f_i(y_i \mid \mathbf{z}; \Theta) =& \log \Gamma(\alpha_i(\mathbf{z}) + \beta_i(\mathbf{z})) - \log \Gamma(\alpha_i(\mathbf{z})) - \log \Gamma(\beta_i(\mathbf{z})) + \\ & (\alpha_i(\mathbf{z}) - 1)\log(y) + (\beta_i(\mathbf{z}) - 1)\log(1-y)\end{aligned}$$

where $\Gamma(\cdot)$ is the gamma function. For simplicity, we drop the functional dependence on $\mathbf{z}$ from the distributional parameters $(\alpha_i, \beta_i)^\intercal$, but it should be clear from context that these distributional parameters depend on $\mathbf{z}$. The first-order derivatives with respect to the shape parameters are:

$$\begin{aligned}\frac{\partial \log f_i(y_i \mid \mathbf{z}; \Theta)}{\partial \alpha_i} &= \psi_0(\alpha_i + \beta_i) - \psi_0(\alpha_i) + \log(y) \\ \frac{\partial \log f_i(y_i \mid \mathbf{z}; \Theta)}{\partial \beta_i} &= \psi_0(\alpha_i + \beta_i) - \psi_0(\beta_i) + \log(1-y)\end{aligned}$$

where $\psi_0(x) = \mathrm{d}\log\Gamma(x)/\mathrm{d}x$ is the digamma function. Aggregate the derivatives above in the vector $\mathbb{F}_{\alpha,\beta} = (\frac{\partial \log f_i(y_i \mid \mathbf{z};\Theta)}{\partial \alpha_i}, \frac{\partial \log f_i(y_i \mid \mathbf{z};\Theta)}{\partial \beta_i})^\intercal$.

The problem with the original parametrization is that α_i and β_i do not have a location-scale interpretation, since both influence the (conditional) mean and (conditional) variance: $\mathbb{E}(y_i \mid \mathbf{z}) = \alpha_i/(\alpha_i + \beta_i)$ and $\mathrm{Var}(y_i \mid \mathbf{z}) = (\alpha_i\beta_i)/(\alpha_i + \beta_i)^2(\alpha_i + \beta_i + 1)$ where both α_i and β_i depend on $\mathbf{z}$.

Instead, in the paper, we follow the alternative parametrization $y_i|\mathbf{z} \sim \mathrm{Beta}(\mu_i(\mathbf{z}), \sigma_i(\mathbf{z}))$, where $\mu_i(\mathbf{z}) \in (0,1)$ is a location parameter and $\sigma_i(\mathbf{z}) \in (0,1)$ is a scale parameter. The relationship between $(\alpha_i, \beta_i)^\intercal$ and $(\mu_i, \sigma_i)^\intercal$ is given by:

$$\mu_i = \frac{\alpha_i}{\alpha_i + \beta_i} \qquad \text{and} \qquad \sigma_i = \frac{1}{(\alpha_i + \beta_i + 1)^{1/2}};$$

with the inverse mappings

$$\alpha_i = \frac{\mu_i(1-\sigma_i^2)}{\sigma_i^2} \qquad \text{and} \qquad \beta_i = \frac{(1-\mu_i)(1-\sigma_i^2)}{\sigma_i^2}.$$

Under this parametrization, $\mathbb{E}(y_i \mid \mathbf{z}) = \mu_i(\mathbf{z})$ and $\mathrm{Var}(y_i \mid \mathbf{z}) = \sigma_i^2(\mathbf{z})\,\mu_i(\mathbf{z})(1 - \mu_i(\mathbf{z}))$.

Let $\mathbb{D}$ be the Jacobian matrix:

$$\mathbb{D} = \begin{bmatrix} \frac{\partial \alpha_i}{\partial \mu_i} & \frac{\partial \alpha_i}{\partial \sigma_i} \\ \frac{\partial \beta_i}{\partial \mu_i} & \frac{\partial \beta_i}{\partial \sigma_i} \end{bmatrix} = \begin{bmatrix} \frac{1}{\sigma_i^2} - 1 & \frac{-2\mu_i}{\sigma_i^3} \\ 1 - \frac{1}{\sigma_i^2} & \frac{2\mu_i - 2}{\sigma_i^3} \end{bmatrix}$$

Then, the vector of first-order derivatives in the location-scale parametrization is $\mathbb{F}_{\mu,\sigma} = (\frac{\partial \log f_i(y_i \mid \mathbf{z};\Theta)}{\partial \mu_i}, \frac{\partial \log f_i(y_i \mid \mathbf{z};\Theta)}{\partial \sigma_i})^\intercal = \mathbb{D}^\intercal \mathbb{F}_{\alpha,\beta}$.

The default link functions for μ_i and σ_i, denoted by $v_{i,\mu}$ and $v_{i,\sigma}$ respectively, are the logit function. Therefore, the derivative of the location parameter μ_i with respect to the linear predictor $\eta_{i,\mu}$ is

$$\frac{\partial \mu_i}{\partial \eta_{i,\mu}} = \frac{\exp(\eta_{i,\mu})}{(1 + \exp(\eta_{i,\mu}))^2},$$

and similarly for σ_i and $\eta_{i,\sigma}$.

With the above, the bracketed expression in (A1.1) becomes

$$\begin{aligned} &\left(\frac{\partial \log f_i(y_i \,|\, \mathbf{z})}{\partial \alpha_i} \frac{\partial \alpha_i}{\partial \mu_i} + \frac{\partial \log f_i(y_i \,|\, \mathbf{z})}{\partial \beta_i} \frac{\partial \beta_i}{\partial \mu_i} \right) \frac{\partial \mu_i}{\partial \eta_{i,\mu}} \frac{\partial \eta_{i,\mu}}{\partial \alpha_{ij,\mu}} \\ &\qquad = \left[\frac{1 - \sigma_i^2}{\sigma_i^2} \cdot (\psi_0(\beta_i) - \psi_0(\alpha_i) + \log(y_i) - \log(1 - y_i)) \cdot \frac{\exp(\eta_{i,\mu})}{(1 + \exp(\eta_{i,\mu}))^2} \right] (1, \mathbf{z})^\intercal \end{aligned} \tag{A1.2}$$

for the vector of factor loadings in the measurement equation for μ_i, and

$$\begin{aligned} &\left(\frac{\partial \log f_i(y_i \,|\, \mathbf{z})}{\partial \alpha_i} \frac{\partial \alpha_i}{\partial \sigma_i} + \frac{\partial \log f_i(y_i \,|\, \mathbf{z})}{\partial \beta_i} \frac{\partial \beta_i}{\partial \sigma_i} \right) \frac{\partial \sigma_i}{\partial \eta_{i,\sigma}} \frac{\partial \eta_{i,\sigma}}{\partial \alpha_{ij,\sigma}} \\ &\qquad = \left[-\frac{2}{\sigma_i^3} \cdot (\mu_i \cdot (\psi_0(\alpha_i + \beta_i) - \psi_0(\alpha_i) + \log(y_i)) + \right. \\ &\qquad\qquad \left. (1 - \mu_i) \cdot (\psi_0(\alpha_i + \beta_i) - \psi_0(\beta_i) + \log(1 - y_i))) \cdot \frac{\exp(\eta_{i,\mu})}{(1 + \exp(\eta_{i,\mu}))^2} \right] (1, \mathbf{z})^\intercal \end{aligned} \tag{A1.3}$$

for the vector of factor loadings in the measurement equation for σ_i.

Skew-Normal Distributed Response Times

Conditional on the latent variables $\mathbf{z}$, let $y_i \,|\, \mathbf{z} \sim \mathrm{SN}(\xi_i(\mathbf{z}), \omega_i(\mathbf{z}), \alpha_i(\mathbf{z}))$ be an observed variable following a Skew-Normal distribution with location parameter $\xi_i(\mathbf{z}) \in \mathbb{R}$, scale parameter $\omega_i(\mathbf{z}) > 0$, and shape (slant) parameter $\alpha_i(\mathbf{z}) \in \mathbb{R}$, for $i = 1, \ldots, p$. The log-density is given by

$$\log f_i(y_i \,|\, \mathbf{z}; \Theta) = -\frac{1}{2}\log(2\pi) - \log(\omega_i) - \frac{(y - \xi_i)^2}{2\omega_i^2} + \zeta_0\left(\alpha_i \frac{y - \xi_i}{\omega_i}\right)$$

where $\zeta_0(\cdot) = \log\{2\Phi(x)\}$ and $\Phi(\cdot)$ is the standard Normal cumulative distribution function.

Let $\tilde{y}_i = (y_i - \xi_i)/\omega_i$, and define $\zeta_1(x) = \mathrm{d}\zeta_0(x)/\mathrm{d}x = \phi(x)/\Phi(x)$, where $\phi(\cdot)$ is the standard Normal density function. The first-order derivatives with respect to the distributional parameters are:

$$\begin{aligned}
\frac{\partial \log f_i(y_i \,|\, \mathbf{z}; \Theta)}{\partial \xi_i} &= \frac{\tilde{y}_i}{\omega_i} - \frac{\alpha_i}{\omega_i}\zeta_1(\alpha_i\, \tilde{y}_i) \\
\frac{\partial \log f_i(y_i \,|\, \mathbf{z}; \Theta)}{\partial \omega_i} &= -\frac{1}{\omega_i} + \frac{\tilde{y}_i^2}{\omega_i} - \frac{\alpha_i}{\omega_i}\zeta_1(\alpha_i\, \tilde{y}_i)\, \tilde{y}_i \\
\frac{\partial \log f_i(y_i \,|\, \mathbf{z}; \Theta)}{\partial \alpha_i} &= \zeta_1(\alpha_i\, \tilde{y}_i)\, \tilde{y}_i
\end{aligned} \tag{A1.4}$$

Define the vector $\mathbb{F}_{\xi,\omega,\alpha} = (\frac{\partial \log f_i(y_i \,|\, \mathbf{z};\Theta)}{\partial \xi_i}, \frac{\partial \log f_i(y_i \,|\, \mathbf{z};\Theta)}{\partial \omega_i}, \frac{\partial \log f_i(y_i \,|\, \mathbf{z};\Theta)}{\partial \alpha_i})^\intercal$ with entries given by the expressions above.

The original parametrization of the SN distribution has two significant limitations. First, the parameters $(\xi_i, \omega_i, \alpha_i)^\intercal$ do not have a direct interpretation in terms of the random variable moments. Second, there is a singularity at $\alpha_i = 0$ that prevents the application of the standard asymptotic theory and leads to convergence issues during the estimation (Azzalini, 2013). The alternative 'centered' parametrization is indexed by the vector of distributional parameters $(\mu_i, \sigma_i, \gamma_i)^\intercal$ given by:

$$\mathbb{E}(y_i \,|\, \mathbf{z}) := \mu_i = \xi_i + b\omega_i\delta_i$$

$$\mathrm{Var}(y_i \,|\, \mathbf{z}) := \sigma_i^2 = \omega_i^2(1 - b^2\delta_i^2)$$

$$\text{Skewness}(y_i \,|\, \mathbf{z}) := \gamma_i \;=\; \frac{4-\pi}{2}\,\frac{b^3\alpha_i^3}{(1+(1-b^2)\alpha_i^2)^{3/2}}\,,$$

where $b = \sqrt{2/\pi}$ and $\delta_i = \alpha_i(1+\alpha_i^2)^{-1/2}$. Here, $\mu_i \in \mathbb{R}$ is a location parameter, $\sigma_i > 0$ is a scale parameter, and $\gamma_i \in (-\tilde{\gamma}, \tilde{\gamma})$ is the skewness index, with $\tilde{\gamma} = \sqrt{2}(4-\pi)\cdot(\pi-2)^{-3/2} \approx 0.9953$ as its upper bound. To model γ_i using latent variables, we apply an additional monotone transformation

$$\nu_i = \frac{\gamma_i + \tilde{\gamma}}{2\,\tilde{\gamma}}$$

that yields a shape parameter $\nu_i \in (0, 1)$.

Let $\mathbb{D}$ be the Jacobian matrix is:

$$\mathbb{D} = \begin{bmatrix} \dfrac{\partial \xi_i}{\partial \mu_i} & \dfrac{\partial \xi_i}{\partial \sigma_i} & \dfrac{\partial \xi_i}{\partial \gamma_i}\dfrac{\mathrm{d}\gamma_i}{\mathrm{d}\nu_i} \\ 0 & \dfrac{\partial \omega_i}{\partial \sigma_i} & \dfrac{\partial \omega_i}{\partial \gamma_i}\dfrac{\mathrm{d}\gamma_i}{\mathrm{d}\nu_i} \\ 0 & 0 & \dfrac{\mathrm{d}\alpha_i}{\mathrm{d}\gamma_i}\dfrac{\mathrm{d}\gamma_i}{\mathrm{d}\nu_i} \end{bmatrix} = \begin{bmatrix} 1 & -b\,\delta_i(1-b^2\delta_i^2)^{-1/2} & \dfrac{-\sigma_i\, b\, \delta_i}{3(1-b^2\delta_i^2)^{1/2}\,\gamma_i}\,2\tilde{\gamma} \\ 0 & (1-b^2\delta_i^2)^{-1/2} & \dfrac{\sigma_i b^2 \delta_i}{(1-b^2\delta_i^2)^{3/2}\cdot(1+\alpha_i^2)^{3/2}}\dfrac{\mathrm{d}\alpha_i}{\mathrm{d}\gamma_i}\,2\tilde{\gamma} \\ 0 & 0 & \dfrac{2}{3(4-\pi)}\left(\dfrac{1}{TR^2} + \dfrac{1-b^2}{T^3}\right)2\tilde{\gamma} \end{bmatrix}, \tag{A1.5}$$

where $T = \left[b^2 - (1-b^2)R^2\right]^{1/2}$ and $R = \sqrt[3]{\frac{2|\gamma_i|}{4-\pi}} \times \text{sign}(\gamma_i)$.

The vector of first-order derivatives in the revised 'centered' parametrization is

$$\mathbb{F}_{\mu,\sigma,\nu} = \left(\frac{\partial \log f_i(y_i \,|\, \mathbf{z}; \Theta)}{\partial \mu_i}, \frac{\partial \log f_i(y_i \,|\, \mathbf{z}; \Theta)}{\partial \sigma_i}, \frac{\partial \log f_i(y_i \,|\, \mathbf{z}; \Theta)}{\partial \nu_i}\right)^{\mathsf{T}} = \mathbb{D}^{\mathsf{T}}\mathbb{F}_{\xi,\omega,\alpha}\,.$$

The default link function for μ_i is the identity link, for σ_i the log link, and ν_i the logit link, and therefore

$$\begin{aligned} \frac{\partial \mu_i}{\partial \eta_{i,\mu}} &= 1 \\ \frac{\partial \sigma_i}{\partial \eta_{i,\sigma}} &= \exp(\eta_{i,\sigma}) \\ \frac{\partial \nu_i}{\partial \eta_{i,\nu}} &= \frac{\exp(\eta_{i,\nu})}{(1+\exp(\eta_{i,\nu}))^2} \end{aligned} \tag{A1.6}$$

The bracketed expression in (A1.1) can be obtained for the vector of factor loadings in the location (μ_i), scale (σ_i), and shape (ν_i) parameters by evaluating the entries in $\mathbb{F}_{\mu,\sigma,\nu}$ using the expressions in (A1.4), the Jacobian matrix (A1.5), and the derivatives for the link functions in (A1.6).

A2. Computation of factor correlations

When $\mathbf{\Phi}$ is a correlation matrix, we need to impose additional restrictions on the parameter space to ensure that the diagonal entries in $\hat{\mathbf{\Phi}}$ are equal to 1. In terms of the Cholesky decomposition $\mathbf{\Phi} = \mathrm{L}\mathrm{L}^{\intercal}$, these restrictions imply that the $j = 1, ..., q$ rows of L, denoted by L_j, satisfy $||\mathrm{L}_j|| = 1$. Computing L becomes a constrained optimization problem, which can be easily handled by an iterative quasi-Newton proximal algorithm (Parikh and Boyd, 2014; Lee et al., 2014; Zhang and Chen, 2022).

Let $\tilde{\mathrm{L}}_j^{[t]}$ be the updated value for row L_j at iteration t of the EM-algorithm or the BFGS solver. $\tilde{\mathrm{L}}_j^{[t]}$ does not satisfy the restriction $||\tilde{\mathrm{L}}_j^{[t]}|| = 1$, and thus we need to project it onto the feasible region of the constrained optimization problem. The proximal update for row L_j simply becomes:

$$\mathrm{L}_j^{[t]} = \underset{\mathrm{L}_j : ||\mathrm{L}_j|| = 1}{\arg\min} ||\mathrm{L}_j - \tilde{\mathrm{L}}_j^{[t+1]}|| = \frac{1}{||\tilde{\mathrm{L}}_j^{[t]}||} \tilde{\mathrm{L}}_j^{[t]}, \qquad \text{for } j = 1, ..., q$$

Zhang and Chen (2022) given a thorough explanation of proximal quasi-Newton algorithms in the context of parameter computation in LVMs.

A note on implementation: The projection step cannot be directly implemented on the BFGS solver in the `R` package `RcppEnsmallen`. Therefore, we propose an alternating procedure in the direct maximization step. First, we obtain $(\hat{\boldsymbol{\alpha}}_0^{\intercal}, \mathrm{vec}(\hat{\mathrm{A}})^{\intercal})$, treating $\hat{\mathbf{\Phi}} = \hat{\mathrm{L}}\hat{\mathrm{L}}^{\intercal}$ (either from the EM-step or previous direct maximization steps) as fixed. Second, we update $\hat{\mathbf{\Phi}}$ while treating $(\hat{\boldsymbol{\alpha}}_0^{\intercal}, \mathrm{vec}(\hat{\mathrm{A}})^{\intercal})$ from the direct maximization step as fixed. We repeat these steps until the marginal log-likelihood converges.

A3. Results on Model Identification

We present key theoretical results on parameter identification of the class of GLVM-LSS models introduced in this paper.

For completeness, we reintroduce the necessary notation and definitions. Let $\mathbf{y} \in \mathbb{R}^p$ represent the observed variables, and $\mathbf{z} \in \mathbb{R}^q$ the latent variables. The score vector contains the first-order derivatives of the marginal log-density of the observed variables with respect to the model parameters, i.e., $\mathbb{S}(\Theta; \mathbf{y}) := \nabla_\Theta \log f(\mathbf{y}; \Theta)$. For LVMs, the score vector entries are given by:

$$\mathbb{S}_k(\Theta; \mathbf{y}) := \frac{\partial}{\partial \theta_k} \log f(\mathbf{y}; \Theta) = \int_{\mathbb{R}^q} \frac{\partial}{\partial \theta_k} \log f(\mathbf{y}, \mathbf{z}; \Theta) \; p(\mathbf{z} \,|\, \mathbf{y}; \Theta) \, \mathrm{d}\mathbf{z} \,, \qquad k = 1, \ldots, K \,,$$

where $f(\mathbf{y}, \mathbf{z}; \Theta) = \prod_{i=1}^{p} f_i(y_i \mid \mathbf{z}; \Theta) p(\mathbf{z}; \Theta)$ is the joint density of observed and latent variables. The domain of $f_i(y_i \mid \mathbf{z}; \Theta)$ is $\mathcal{D}_i \subseteq \mathbb{R}$, and $\mathcal{D} = \mathcal{D}_1 \times \cdots \times \mathcal{D}_p \subseteq \mathbb{R}^p$ is the domain of $\mathbf{y}$. The parameter space is $\Xi \subseteq \mathbb{R}^K$.

Model identifiability is crucial for making statistical inferences about model parameters. We introduce the following definitions:

Definition A3.1. *Two parameter points, Θ_0 and Θ_1 in Ξ are observationally equivalent if $f(\mathbf{y}; \Theta_0) = f(\mathbf{y}; \Theta_1)$, for all $\mathbf{y} \in \mathcal{D}$.*

Definition A3.2. *A parameter point $\Theta_0 \in \Xi$ is globally identifiable if no other $\Theta \in \Xi$ is observationally equivalent to Θ_0.*

Global identification conditions often depend on specific model structures and are analytically difficult to verify (Shapiro, 1985). For general models, including GLLVMs, we consider the weaker concept of local identification (Skrondal and Rabe-Hesketh, 2004, Chapter 5):

Definition A3.3. *A parameter point Θ_0 is locally identifiable if there exists an open neighborhood around Θ_0 that contains no other point observationally equivalent to Θ_0.*

The expected information matrix, $\mathcal{I}(\Theta) := \mathbf{E}_{\mathbf{y}}[\mathbb{S}(\Theta; \mathbf{y})\mathbb{S}(\Theta; \mathbf{y})^\intercal]$, is fundamental for assessing local identifiability. We denote the expected information matrix and score vector

evaluated at $\Theta_0 \in \Xi$ by $\mathcal{I}(\Theta_0)$ and $\mathbb{S}(\Theta_0; \mathbf{y})$, respectively. The concept of a regular point is also important:

Definition A3.4. *Let* $\mathrm{M}(\Theta)$ *be a continuous matrix-valued function of* $\Theta \in \Xi$*. A point* $\Theta_0 \in \Xi$ *is a regular point of* $\mathrm{M}(\Theta)$ *if the rank of* $\mathrm{M}(\Theta_0)$ *remains constant in an open neighborhood around* Θ_0*.*

The following result is presented as a lemma, but its importance in model identification stems from the seminal theorem in Rothenberg (1971):

Lemma A3.1 (Theorem 1 in Rothenberg, 1971)**.** *Let* $\Theta_0 \in \Xi$ *be a regular point of* $\mathcal{I}(\Theta)$*. Assume* $f(\mathbf{y}; \Theta) \in \mathrm{C}^1(\Xi)$ *and* $\log f(\mathbf{y}; \Theta) \in \mathrm{C}^1(\Xi)$ *for all* $\mathbf{y} \in \mathcal{D}$*. Then* Θ_0 *is locally identifiable if, and only if,* $\mathcal{I}(\Theta_0)$ *is positive-definite.*

Proof of Lemma A3.1: See Rothenberg (1971) or Bekker et al. (1994, Chapter 2). □

Lemma A3.1 relates to the idea of *strict identifiability*, a point-wise condition for a given $\Theta_0 \in \Xi$. In the GLLVM, (strict) local identifiability is only possible for points on the reduced parameter space that results from imposing q^2 restrictions across the factor loadings matrix A and the latent variables covariance matrix $\mathbf{\Phi}$. These restrictions address rotational indeterminacy and fix the scale of the latent variable space (Anderson and Rubin, 1956). Unless stated otherwise, when referring to a parameter point $\Theta_0 \in \Xi$, we refer to a point in the *reduced* parameter space.

Strict identifiability can be restrictive, as it applies only to a specific point in the parameter space, ignoring other $\Theta \in \Xi$ that may also be locally identifiable almost surely. In this paper, we adopt the concept of 'generic identifiability' (Allman et al., 2009), which ensures identifiability almost everywhere in the parameter space, except for a set $V = \{\Theta \in \Xi : p_i(\Theta) = 0\,, 1 \leq i \leq P\}$, where $\{p_i\}_{i=1}^P$ is a finite set of multivariate polynomials. If $\dim(V) < \dim(\Xi)$, V is called a *proper sub-variety* of Ξ and has Lebesgue measure zero in Ξ (see, e.g., Gu and Xu, 2020). For a generically identified model, parameter inference is valid for 'generic' points $\Theta \in \Xi \setminus V$. In particular, we adopt the idea of generic *local* identifiability.

Definition A3.5. *A statistical model is generically locally identified on* $\Xi \subseteq \mathbb{R}^K$ *if the parameters* Θ *are locally identifiable on* $\Xi \setminus V$*, where* V *is a proper sub-variety of* Ξ*.*

Studying the generic local identifiability of the GLVM-LSS is beneficial for two reasons. First, as mentioned above, strict identifiability is a restrictive, point-wise condition. Second, the presence of multiple distributional parameters might complicate model identifiability, especially when the location, scale, and shape parameters are correlated.

We prove the generic local identifiability of the GLVM-LSS models with continuous observed variables by examining the relationship between the rank of $\mathcal{I}(\Theta)$ and the linear independence of the entries of the score vector $\mathbb{S}(\Theta; \mathbf{y})$ for $\Theta \in \Xi \setminus V$. In what follows, we assume $\Xi = \mathbb{R}^K$ without loss of generality. The next proposition is useful:

Proposition A3.1. *Let $\Theta_0 \in \mathbb{R}^K$ be a fixed parameter point. Then, $\mathcal{I}(\Theta_0)$ is positive-definite if, and only if, all entries of $\mathbb{S}(\Theta_0; \mathbf{y})$, taken as functions of $\mathbf{y}$, are linearly independent for all $\mathbf{y} \in \mathcal{D}$ such that $f(\mathbf{y}; \Theta) > 0$ in an open neighborhood of Θ_0.*

Proof of Proposition A3.1: ($\Longrightarrow$) From standard likelihood theory we know that $\mathrm{Var}_{\mathbf{y}}[\mathbb{S}(\Theta; \mathbf{y})] = \mathcal{I}(\Theta)$. Suppose $\mathcal{I}(\Theta_0)$ is not positive-definite for some $\Theta_0 \in \mathbb{R}^K$. Then, there exists a vector $\boldsymbol{\upsilon} \in \mathbb{R}^K \setminus \{\mathbf{0}\}$ such that:

$$0 = \boldsymbol{\upsilon}^\intercal \, \mathrm{Var}_{\mathbf{y}}[\mathbb{S}(\Theta_0; \mathbf{y})] \, \boldsymbol{\upsilon} = \mathrm{Var}_{\mathbf{y}}[\boldsymbol{\upsilon}^\intercal \, \mathbb{S}(\Theta_0; \mathbf{y})] \, ,$$

indicating that the non-trivial linear combination $\boldsymbol{\upsilon}^\intercal \mathbb{S}(\Theta_0; \mathbf{y})$ has zero variance and is constant almost surely. Defining $\mathrm{B} := \{\mathbf{X} \in \mathbb{R}^K : \boldsymbol{\upsilon}^\intercal \mathbf{X} = \beta\}$ for some arbitrary constant β, we have $\mathbb{P}(\mathbb{S}(\Theta_0; \mathbf{y}) \in \mathrm{B}) = 1$, implying the entries of $\mathbb{S}(\Theta_0; \mathbf{y})$ are linearly dependent.

($\Longleftarrow$) Now assume the entries of $\mathbb{S}(\Theta_0; \mathbf{y})$ are linearly dependent. Thus, without loss of generality, we can write $\mathbb{S}(\Theta_0; \mathbf{y}) = \mathrm{A} Z(\Theta_0; \mathbf{y})$, where $\mathrm{A} \in \mathbb{R}^{K \times h}$ with $h \ll K$ is a matrix of fixed coefficients, and $Z(\Theta_0; \mathbf{y}) \in \mathbb{R}^h$ is a random vector with $\mathbb{E}_{\mathbf{y}}(Z(\Theta_0; \mathbf{y}) Z(\Theta_0; \mathbf{y})^\intercal)$ full rank. Then,

$$\mathcal{I}(\Theta_0) = \mathrm{Var}_{\mathbf{y}}[\mathbb{S}(\Theta_0; \mathbf{y})] = \mathrm{A} \mathbf{E}_{\mathbf{y}}[Z(\Theta_0; \mathbf{y}) \, Z(\Theta_0; \mathbf{y})^\intercal] \mathrm{A}^\intercal$$

which has rank at most h and is not positive definite. This completes the proof of Proposition A3.1. □

We now present the main theorem on the generic local identifiability of the GLVM-LSS models introduced in this paper:

Theorem A3.1. *Assume:*

(A1) There exists a point in the reduced parameter space $\Theta_0 \in \mathbb{R}^K$ *such that* $\mathcal{I}(\Theta_0)$ *is strictly positive definite,*

(A2) $f_i(y_i \,|\, \mathbf{z}; \boldsymbol{\theta}_i(\Theta))$, $i = 1, \ldots, p$, *in the measurement part, and* $p(\mathbf{z}; \Theta)$ *in the structural part of a GLVM-LSS model, are infinitely differentiable in* $\mathbb{R}^K$ *and* $\mathcal{D}$*, and their respective supports are independent of* Θ*,*

Then, the GLVM-LSS model is generically locally identified for every $\Theta \in \mathbb{R}^K \setminus V$*, where* V *is a set of Lebesgue measure zero.*

Before starting with the proof, we explain assumption (A1) in Theorem A3.1. This assumption prevents trivial non-identification issues, such as rotational or scale indeterminacies. Indeed, imposing identifiability restrictions on the model parameters ensures that at least one point in the reduced parameter space is strictly locally identified. Without addressing these non-identification issues, the Fisher information matrix would be rank-deficient for all points in the parameter space. This assumption is easy to verify in practice, e.g., by checking the expected information matrix is full-rank at the MLE. It also precludes cases when distributional parameters are linearly dependent.

Proof of Theorem A3.1: The proof of Theorem A3.1 relies on tools from real and complex analysis and is divided into several lemmas, some of independent interest.

The following is an outline of the proof: Under the assumptions and smoothness and regularity conditions on $f(\mathbf{y} \,|\, \mathbf{z}; \Theta)$ and $p(\mathbf{z}; \Theta)$ mentioned in the theorem, we show that the entries of the score vector $\mathbb{S}(\Theta; \mathbf{y})$ are linearly independent for all $\mathbf{y} \in \mathcal{D}$ such that $f(\mathbf{y}; \Theta) > 0$, and for all $\Theta \in \mathbb{R}^K \setminus V$, where V is a set of Lebesgue measure zero. By Proposition A3.1, we have that $\mathcal{I}(\Theta)$ is positive definite for all $\Theta \in \mathbb{R}^K \setminus V$, and thus, by Lemma A3.1 and the definition of generic local identifiability, the GLVM-LSS is generically locally identified for $\Theta \in \mathbb{R}^K \setminus V$.

We define the family of functions $g_k : \mathcal{D} \times \mathbb{R}^K \to \mathbb{R}$ as $g_k(\mathbf{y}, \Theta) := \mathbb{S}_k(\Theta; \mathbf{y})$, and express:

$$g_k(\mathbf{y}, \Theta) = \int_{\mathbb{R}^q} g_k^a(\mathbf{y}, \Theta; \mathbf{z})\, g_k^b(\mathbf{y}, \Theta; \mathbf{z})\; \mathrm{d}\mathbf{z}\,, \qquad \text{for} \quad k = 1, \ldots, K\,,$$

where

$$g_k^a(\mathbf{y},\Theta;\mathbf{z}) := \frac{\partial}{\partial\theta_k}\log f(\mathbf{y},\mathbf{z};\Theta) = \sum_{i=1}^{p}\frac{\partial}{\partial\theta_k}\log f_i(y_i\,|\,\mathbf{z};\Theta) + \frac{\partial}{\partial\theta_k}\log p(\mathbf{z};\Theta)$$

and

$$g_k^b(\mathbf{y},\Theta;\mathbf{z}) := p(\mathbf{z}\,|\,\mathbf{y};\Theta)\,.$$

The following lemma helps establish the linear independence of the entries of the score vector:

Lemma A3.2. *The functions $(\mathbf{y},\Theta)\mapsto g_k(\mathbf{y},\Theta)$, $k=1,\ldots,K$, are real-analytic on an open set of $\mathcal{D}\times\mathbb{R}^K$.*

To prove Lemma A3.2, we use the concept of separate-analytic functions:

Definition A3.6. *A function $h : A\times B\to\mathbb{R}$, $A\subseteq\mathbb{R}^a$, $B\subseteq\mathbb{R}^b$, is called separately real-analytic on $A\times B$ if $y\mapsto h(x,y)$ is real-analytic on B for each $x\in A$, and $x\mapsto h(x,y)$ is real-analytic on A for each $y\in B$.*

We introduce the following notation. For a function $h : A\to\mathbb{R}$ and $x=(x_1,\ldots,x_a)\in A$, we define the partial derivative with respect to x_i as:

$$D_i h = \frac{\partial}{\partial x_i}h \qquad \text{for} \quad 1\le i\le a\,.$$

Using the multi-index notation, we write

$$D^\alpha = \prod_{i=1}^{a} D_i^{\alpha_i} = \frac{\partial^{\alpha_1}}{(\partial x_1)^{\alpha_1}}\frac{\partial^{\alpha_2}}{(\partial x_2)^{\alpha_2}}\cdots\frac{\partial^{\alpha_a}}{(\partial x_a)^{\alpha_a}}\,,$$

for any a-tuple $(\alpha_1,\ldots,\alpha_a)$ of non-negative integers. Additionally, we define

$$\alpha = \sum_{i=1}^{a}\alpha_i \qquad \text{and} \qquad \alpha! = \prod_{i=1}^{a}(\alpha_i)!\,.$$

For a function $h : A\times B\to\mathbb{R}$, we use D_x^α and D_y^α to denote the derivatives with respect to $x\in A$ and $y\in B$, respectively.

The proof of Lemma A3.2 also relies on Lemma A3.3 (a result from complex analysis) and Proposition A3.2:

Lemma A3.3 (Theorem 2 in Browder, 1961)**.** *Let h be infinitely differentiable and separately analytic on $\mathcal{D}\times\mathbb{R}^K$. Then there exists an everywhere dense open subset of $\mathcal{D}\times\mathbb{R}^K$ where h is jointly analytic.*

Proof of Lemma A3.3: Browder (1961), Krantz and Parks (2002, Section 4.3), □

Proposition A3.2. *Assume the following regularity and smoothness conditions:*

(C1) The support of $\mathbf{y}$, $\mathcal{Y}=\{\mathbf{y}\in\mathcal{D}: f(\mathbf{y}\,|\,\mathbf{z};\Theta)>0\}$, is independent of Θ.

(C2) For all $\Theta\in\mathbb{R}^K$, $\mathbf{y}\mapsto f(\mathbf{y}\,|\,\mathbf{z};\Theta)$ is infinitely differentiable on $\mathcal{D}$. Similarly, for all $\mathbf{y}\in\mathcal{D}$, $\Theta\mapsto f(\mathbf{y}\,|\,\mathbf{z};\Theta)$ and $\Theta\mapsto p(\mathbf{z};\Theta)$ are infinitely differentiable on $\mathbb{R}^K$.

(C3) For fixed $\bar{\Theta}\in\mathbb{R}^K$ and for all $\mathbf{y}$ in a compact subset of $\mathcal{D}$, $\mathbf{y}\mapsto f(\mathbf{y}\,|\,\mathbf{z};\bar{\Theta})$ admits an analytical continuation on an open subset of $\mathbb{C}^p$ and is bounded. Similarly, for fixed $\bar{\mathbf{y}}\in\mathcal{D}$ and for all Θ in a compact subset of $\mathbb{R}^K$, the mappings $\Theta\mapsto f(\bar{\mathbf{y}}\,|\,\mathbf{z};\Theta)$ and $\Theta\mapsto p(\mathbf{z};\Theta)$ admit analytical continuations on open subsets of $\mathbb{C}^K$ and are bounded.

Under these conditions, $f(\mathbf{y}\,|\,\mathbf{z};\Theta)$ and $p(\mathbf{z};\Theta)$ are jointly real-analytic on an open subset of $\mathcal{D}\times\mathbb{R}^K$.

Proof of Proposition A3.2: Using Lemma A3.3, we need to show: i) $f(\mathbf{y}\,|\,\mathbf{z};\Theta)\in C^\infty(\mathcal{D}\times\mathbb{R}^K)$ and $p(\mathbf{z};\Theta)\in C^\infty(\mathcal{D}\times\mathbb{R}^K)$; and ii) that $f(\mathbf{y}|\mathbf{z};\Theta)$ and $p(\mathbf{z};\Theta)$ are separately real-analytic on an open subset of $\mathcal{D}\times\mathbb{R}^K$.

i) Joint smoothness on $\mathcal{D}\times\mathbb{R}^K$: For completeness, we summarize the proofs in Krantz and Parks (2002, Lemma 4.3.4) and Browder (1961), referring to them for details.

Given conditions (C2) and (C3) –following assumption (A2) in Theorem A3.1–, the Cauchy integral formula for poly-cylinders in an open subset of $\mathcal{D}\times\mathbb{R}^K$ (embedded in $\mathbb{C}^p\times\mathbb{C}^K$) gives a constant $C_0>0$ such that, for all α and β:

$$|D^\alpha_{\mathbf{y}}f(\mathbf{y}\,|\,\mathbf{z};\Theta)|\le C_0^\alpha\alpha! \qquad \text{and} \qquad |D^\alpha_{\mathbf{y}}p(\mathbf{z};\Theta)|\le C_0^\alpha\alpha!\,,$$
$$|D^\beta_{\Theta}f(\mathbf{y}\,|\,\mathbf{z};\Theta)|\le C_0^\beta\beta! \qquad \text{and} \qquad |D^\beta_{\Theta}p(\mathbf{z};\Theta)|\le C_0^\beta\beta!\,.$$

hold for all $(\mathbf{y},\Theta)$ in the open subset of $\mathcal{D}\times\mathbb{R}^K$. Since $f(\mathbf{y}|\mathbf{z};\Theta)$ and $p(\mathbf{z};\Theta)$ are separately continuous and thus measurable (Johnson, 1969), their partial derivatives coincide with

distributional derivatives. Therefore, for any integer $r \geq 0$:

$$\left(\sum_{i=1}^{p} D_{y_i}^{2r} + \sum_{k=1}^{K} D_{\theta_k}^{2r}\right) f(\mathbf{y} \,|\, \mathbf{z}; \Theta) \qquad \text{and} \qquad \left(\sum_{i=1}^{p} D_{y_i}^{2r} + \sum_{k=1}^{K} D_{\theta_k}^{2r}\right) p(\mathbf{z}; \Theta)$$

are bounded. By standard regularity results for elliptic partial differential equations (see, e.g., Bets et al., 1963), we conclude that $D_{\mathbf{y}}^{\alpha} D_{\Theta}^{\beta} f(\mathbf{y} \,|\, \mathbf{z}; \Theta) \in \mathrm{L}^2_{loc}$ and $D_{\mathbf{y}}^{\alpha} D_{\Theta}^{\beta} p(\mathbf{z}; \Theta) \in \mathrm{L}^2_{loc}$ for all α and β. By the Sobolev Embedding Theorem, this implies $f(\mathbf{y} \,|\, \mathbf{z}; \Theta) \in C^{\infty}(\mathcal{D} \times \mathbb{R}^K)$ and $p(\mathbf{z}; \Theta) \in C^{\infty}(\mathcal{D} \times \mathbb{R}^K)$.

Next, before proving the separate analyticity of $f(\mathbf{y}|\mathbf{z}; \Theta)$ and $p(\mathbf{z}; \Theta)$ on $\mathcal{D} \times \mathbb{R}^K$, recall that real-analytic functions are closed under addition, multiplication, division (when the denominator is non-zero), derivation, integration, and composition (Krantz and Parks, 2002). Functions like log and exp are also real-analytic on their domains.

ii.a) Separate analyticity in y: Fix $\bar{\Theta} \in \mathbb{R}^K$. The mapping $\mathbf{y} \mapsto f(\mathbf{y} \,|\, \mathbf{z}; \bar{\Theta})$ is real-analytic on an open subset of $\mathcal{D}$ if each $y_i \mapsto f_i(y_i \,|\, \mathbf{z}; \bar{\Theta})$, $i = 1, \ldots, p$, is real-analytic on an open subset of $\mathcal{D}_i$. This typically holds under standard regularity conditions (e.g., Lehmann and Casella, 1998) for common parametric forms of f_i.

For example, if f_i is a continuous distribution in the exponential family, indexed by the vector of distributional parameters $\boldsymbol{\theta}_i$, we have:

$$f_i(y_i; \bar{\Theta}, \mathbf{z}) = h(y_i) \exp(\eta(\boldsymbol{\theta}_i(\bar{\Theta}, \mathbf{z}))^{\intercal} \mathbf{T}(y_i) - A(\boldsymbol{\theta}_i(\bar{\Theta}, \mathbf{z}))) \,,$$

where $h(y_i)$ is a normalizing factor and $\mathbf{T}(y_i)$ a vector of sufficient statistics. We write $\boldsymbol{\theta}_i(\bar{\Theta}, \mathbf{z})$ to capture the dependence on the model parameters and latent variables.

If both $h(y_i)$ and $\exp(\eta(\boldsymbol{\theta}_i(\bar{\Theta}, \mathbf{z}))^{\intercal} \mathbf{T}(y_i))$ are real-analytic, then $y_i \mapsto f_i(y_i \,|\, \mathbf{z}; \bar{\Theta})$ is real-analytic. In the exponential family, $h(y_i)$ often consists of constants, polynomials, or smooth elementary functions that admit analytic continuations. Additionally, $\mathbf{T}(y_i)$ is typically linear, polynomial, or another smooth real-analytic function (e.g., 'log'). By composition, $\exp(\eta(\boldsymbol{\theta}_i(\bar{\Theta}, \mathbf{z}))^{\intercal} \mathbf{T}(y_i))$ is analytic in y_i. Therefore, for common continuous distributions in the exponential family, $y_i \mapsto f_i(y_i|\mathbf{z}; \bar{\Theta})$ is real-analytic on an open subset of $\mathcal{D}_i$.

For completeness, we provide two additional examples of continuous distributions used in this paper: the Skew-Normal (SN) distribution (Azzalini, 2013) and the location-scale parametrization of the Beta distribution (Rigby et al., 2020), described in Appendix A1.

The (conditional) density of a SN-distributed random variable y_i is indexed by the vector of location, scale, and shape parameters $\boldsymbol{\theta}_i = (\xi_i, \omega_i, \alpha_i)^\intercal$, all functions of $(\bar{\Theta}, \mathbf{z})$ but omitted for ease of notation. The SN density given by:

$$f_i(y_i; \bar{\Theta}, \mathbf{z}) = \frac{2}{\omega_i} \phi\left(\frac{y_i - \xi_i}{\omega_i}\right) \Phi\left(\alpha_i \frac{y_i - \xi_i}{\omega_i}\right),$$

where $\phi(x)$ and $\Phi(x)$ are the standard Normal density and cumulative distribution functions, respectively, the latter commonly expressed in terms of the error function $\mathrm{erf}(x)$, $\Phi(x) = \frac{1}{2}[1 + \mathrm{erf}(x/\sqrt{2})]$. The mapping $y_i \mapsto f_i(y_i \mid \mathbf{z}; \bar{\Theta})$ is analytic in y_i because: i) $\frac{y_i - \xi_i}{\omega_i}$ and $\alpha_i \frac{y_i - \xi_i}{\omega_i}$ are linear in y_i, ii) 'exp' and 'erf' are real-analytic, and iii) $\phi(\cdot)$ and $\Phi(\cdot)$ are analytic by composition.

For the Beta distribution, indexed by the vector location and scale parameters $\boldsymbol{\theta}_i = (\mu_i, \sigma_i)^\intercal$, also functions of $(\bar{\Theta}, \mathbf{z})$ but omitted for simplicity, the density function is:

$$f_i(y_i; \bar{\Theta}, \mathbf{z}) = \frac{1}{\mathrm{B}(\alpha_i, \beta_i)} y_i^{\alpha_i - 1} (1 - y_i)^{\beta_i - 1},$$

where $\alpha_i = \mu_i(1 - \sigma_i^2)/\sigma_i^2$ and $\beta_i = (1 - \mu_i)(1 - \sigma_i^2)/\sigma_i^2$. Since the terms $y_i^{\alpha_i - 1}$ and $(1 - y_i)^{\beta_i - 1}$ are analytic on $(0, 1)$ for $\alpha_i, \beta_i > 0$, $y_i \mapsto f_i(y_i \mid \mathbf{z}; \bar{\Theta})$ is analytic for the Beta distribution.

Lastly, since $p(\mathbf{z}; \bar{\Theta})$ does not depend on $\mathbf{y}$, it is constant on $\mathcal{D}$. As constant functions are real-analytic, the mapping $\mathbf{y} \mapsto p(\mathbf{z}; \bar{\Theta})$ is real-analytic on $\mathcal{D}$.

ii.b) Separate analyticity in Θ: Fix $\bar{\mathbf{y}} \in \mathcal{D}$. The mapping $\Theta \mapsto f(\bar{\mathbf{y}} \mid \mathbf{z}; \Theta)$ is real-analytic on an open subset of $\mathbb{R}^K$ if each $\Theta \mapsto f_i(\bar{y}_i \mid \mathbf{z}; \Theta)$, $i = 1, \ldots, p$, is real-analytic.

For continuous distributions f_i in the exponential family, it suffices to show that the natural parameters $\eta(\boldsymbol{\theta}_i(\Theta))$ and the log-partition function $A(\boldsymbol{\theta}_i(\Theta))$ are analytic in Θ. The parameters $\boldsymbol{\theta}_i(\Theta)$ depend on Θ via monotonic differentiable link functions (e.g., log, logit), making $\boldsymbol{\theta}_i(\Theta)$ analytic. The natural parameters $\eta(\boldsymbol{\theta}_i(\Theta))$ are typically linear, polynomial, or smooth in $\boldsymbol{\theta}_i$, so $\eta(\boldsymbol{\theta}_i(\Theta))$ is also analytic. The log-partition function

$A(\boldsymbol{\theta}_i(\Theta))$ is analytic if the integral defining it is well-behaved, which holds for common continuous exponential family distributions. Hence, $\Theta \mapsto f_i(\bar{y}_i \,|\, \mathbf{z}; \Theta)$ is real-analytic.

For the SN distribution, $\Theta \mapsto f_i(\bar{y}_i|\mathbf{z}; \Theta)$ is analytic because: i) $(\xi_i, \omega_i, \alpha_i)$ are analytic in Θ through appropriate link functions, ii) $\frac{y_i - \xi_i}{\omega_i}$ and $\alpha_i \frac{y_i-\xi_i}{\omega_i}$ are linear in $(\xi_i, \omega_i, \alpha_i)$, iii) 'exp' and 'erf' are real-analytic, and iv) by composition, $\phi(\frac{y_i-\xi_i}{\omega_i})$ and $\Phi(\alpha_i \frac{y_i-\xi_i}{\omega_i})$ are real-analytic in Θ.

For the Beta distribution, i) α_i and β_i are analytic in Θ (through μ_i and σ_i), ii) $B(\alpha_i, \beta_i)$ is analytic for $\alpha_i, \beta_i > 0$, and iii) the polynomial terms $y_i^{\alpha_i - 1}$ and $(1 - y_i)^{\beta_i - 1}$ are analytic. Thus, $\Theta \mapsto f_i(\bar{y}_i \,|\, \mathbf{z}; \Theta)$ is analytic for the Beta distribution.

Finally, $\Theta \mapsto p(\mathbf{z}; \Theta)$ is analytic since we assume $\mathbf{z} \sim \mathbb{N}(\mathbf{0}, \Sigma(\Theta))$, and the multivariate Normal distribution is analytic in its parameters.

ii) Joint analyticity in $(\mathbf{y}, \Theta)$: By Lemma A3.3, along with the facts that $f(\mathbf{y}|\mathbf{z};\Theta) \in \mathrm{C}^\infty(\mathcal{D} \times \mathbb{R}^K)$ and $p(\mathbf{z};\Theta) \in \mathrm{C}^\infty(\mathcal{D} \times \mathbb{R}^K)$; and that both are separately analytic in $(\mathbf{y}, \Theta)$, we conclude the proof of Proposition A3.2. □

We are now ready to prove Lemma A3.2.

Proof of Lemma A3.2: By Proposition A3.2, $f(\mathbf{y} \,|\, \mathbf{z}; \Theta)$ and $p(\mathbf{z}; \Theta)$ are jointly real-analytic in an open subset of $\mathcal{D} \times \mathbb{R}^K$. Since the derivatives and 'log' of real-analytic functions remain real-analytic, $\frac{\partial}{\partial \theta_k} \log f_i(y_i \,|\, \mathbf{z}; \Theta)$ and $\frac{\partial}{\partial \theta_k} \log p(\mathbf{z}; \Theta)$ are also jointly real-analytic. Therefore, $(\mathbf{y}, \Theta) \mapsto g_k^a(\mathbf{y}, \Theta)$ is jointly real-analytic.

The posterior distribution of the latent variables,

$$p(\mathbf{z} \,|\, \mathbf{y}; \Theta) = \frac{\prod_{i=1}^{p} f_i(y_i \,|\, \mathbf{z}; \Theta)\, p(\mathbf{z}; \Theta)}{\int_{\mathbb{R}^q} \prod_{i=1}^{p} f_i(y_i \,|\, \mathbf{z}; \Theta)\, p(\mathbf{z}; \Theta)\; \mathrm{d}\mathbf{z}}\,,$$

depends on $f(\mathbf{y}|\mathbf{z};\Theta)$ and $p(\mathbf{z};\Theta)$, both real-analytic in $(\mathbf{y}, \Theta)$. Hence, $(\mathbf{y}, \Theta) \mapsto g_k^b(\mathbf{y}; \mathbf{z}, \Theta)$ is jointly real-analytic as well.

Since the integral over $\mathbf{z}$ is well-behaved, $(\mathbf{y}, \Theta) \mapsto g_k(\mathbf{y}, \Theta)$ is real-analytic for all $(\mathbf{y}, \Theta)$ in the open subset of $\mathcal{D} \times \mathbb{R}^K$. This completes the proof of Lemma A3.2. □

We now continue with the proof of Theorem A3.1. Since the functions $g_k(\mathbf{y};\Theta)$, $k = 1,\ldots,K$, are jointly real-analytic on an open subset of $\mathcal{D}\times\mathbb{R}^K$ (by Lemma A3.2), for a fixed $\Theta_0 \in \mathbb{R}^K$ satisfying assumption (A1), the mappings $\mathbf{y} \mapsto g_k(\mathbf{y};\Theta_0)$ are real-analytic in an open subset of $\mathcal{D}$. Thus, they admit a Taylor expansion around some $\mathbf{y}_0 \in \mathcal{D}$ such that $f(\mathbf{y}_0;\Theta) > 0$ for all Θ in a neighborhood of Θ_0. Using the first K^{th}-order terms, we approximate:

$$
\begin{aligned}
g_k(\mathbf{y};\Theta_0) \approx\ & g_k(\mathbf{y}_0;\Theta_0) \\
& + \sum_{i_1=1}^{p} \frac{\partial g_k(\mathbf{y}_0;\Theta_0)}{\partial y_{i_1}}(y_{i_1}-y_{0i_1}) \\
& + \frac{1}{2}\sum_{i_1=1}^{p}\sum_{i_2=1}^{p} \frac{\partial^2 g_k(\mathbf{y}_0;\Theta_0)}{\partial y_{i_1}\partial y_{i_2}}(y_{i_1}-y_{0i_1})(y_{i_2}-y_{0i_2}) \\
& + \frac{1}{6}\sum_{i_1=1}^{p}\sum_{i_2=1}^{p}\sum_{i_3=1}^{p} \frac{\partial^3 g_k(\mathbf{y}_0;\Theta_0)}{\partial y_{i_1}\partial y_{i_2}\partial y_{i_3}}(y_{i_1}-y_{0i_1})(y_{i_2}-y_{0i_2})(y_{i_3}-y_{0i_3}) \\
& \cdots \\
& + \frac{1}{K!}\sum_{i_1=1}^{p}\cdots\sum_{i_K=1}^{p} \frac{\partial^K g_k(\mathbf{y}_0;\Theta_0)}{\partial y_{i_1}\cdots\partial y_{i_K}}(y_{i_1}-y_{0i_1})\cdots(y_{i_K}-y_{0i_K}),
\end{aligned}
$$

with equality holding up to a residual term of order $o(||\mathbf{y}-\mathbf{y}_0||^K)$.

Thus, the score vector (as a function of $\mathbf{y}$ with fixed $\Theta_0 \in \mathbb{R}^K$) can be written as the product of a $K \times \sum_{k=0}^{K} p^k$ matrix and a $\sum_{k=0}^{K} p^k \times 1$ vector (without loss of generality, let $K \ll \sum_{k=0}^{K} p^k$):

$$
\mathbb{S}(\Theta_0;\mathbf{y}) =: \begin{bmatrix} g_1(\mathbf{y};\Theta_0) \\ g_2(\mathbf{y};\Theta_0) \\ \vdots \\ g_K(\mathbf{y};\Theta_0) \end{bmatrix} \approx \begin{bmatrix} g_1 & \frac{\partial g_1}{\partial y_1} & \cdots & \frac{\partial g_1}{\partial y_p} & \frac{\partial^2 g_1}{(\partial y_1)^2} & \frac{\partial^2 g_1}{\partial y_1 \partial y_2} & \frac{\partial^2 g_1}{\partial y_1 \partial y_3} & \cdots & \frac{\partial^K g_1}{(\partial y_p)^K} \\ g_2 & \frac{\partial g_2}{\partial y_1} & \cdots & \frac{\partial g_2}{\partial y_p} & \frac{\partial^2 g_2}{(\partial y_1)^2} & \frac{\partial^2 g_2}{\partial y_1 \partial y_2} & \frac{\partial^2 g_2}{\partial y_1 \partial y_3} & \cdots & \frac{\partial^K g_2}{(\partial y_p)^K} \\ \vdots & \vdots & \vdots & \vdots & \vdots & \vdots & \vdots & \vdots & \vdots \\ g_K & \frac{\partial g_K}{\partial y_1} & \cdots & \frac{\partial g_K}{\partial y_p} & \frac{\partial^2 g_K}{(\partial y_1)^2} & \frac{\partial^2 g_K}{\partial y_1 \partial y_2} & \frac{\partial^2 g_K}{\partial y_1 \partial y_3} & \cdots & \frac{\partial^K g_K}{(\partial y_p)^K} \end{bmatrix} \begin{bmatrix} 1 \\ y_1 - y_{0,1} \\ \vdots \\ y_p - y_{0,p} \\ \frac{(y_1-y_{0,1})^2}{2} \\ \frac{(y_1-y_{0,1})(y_2-y_{0,2})}{2} \\ \frac{(y_1-y_{0,1})(y_3-y_{0,3})}{2} \\ \vdots \\ \frac{(y_p-y_{0,p})^K}{K!} \end{bmatrix}
$$

$$
= \mathrm{G}(\mathbf{y}_0;\Theta_0)\,\mathbf{v}_K
$$

where $\mathrm{G}(\mathbf{y}_0;\Theta_0)$ is the result of the matrix-valued function $G : \mathcal{D} \times \mathbb{R}^K \to \mathbb{R}^{K \times \sum_{k=0}^{K} p^k}$, evaluated at $(\mathbf{y}_0;\Theta_0)$, with rows composed of the partial derivatives of g_k up to order K. The vector $\mathbf{v}_K$ contains polynomial terms of $(\mathbf{y} - \mathbf{y}_0)$ up to order K. Then, following the analytic properties of $g_k(\mathbf{y};\Theta)$, $k = 1, \ldots, K$, the entries of $\mathrm{G}(\mathbf{y};\Theta)$ are jointly real-analytic in an open subset of $\mathcal{D} \times \mathbb{R}^K$.

Consider the matrix-valued function $\Theta \mapsto \mathrm{G}(\mathbf{y}_0, \Theta)$, with real-analytic entries on an open subset of $\mathbb{R}^K$, for fixed $\mathbf{y}_0 \in \mathcal{D}$ such that $f(\mathbf{y}_0;\Theta) > 0$ locally for all Θ in this subset. For simplicity, we write $\mathrm{G}_{\mathbf{y}_0}(\Theta) := \mathrm{G}(\mathbf{y}_0;\Theta)$. Define: i) the matrix-valued function $\mathrm{M}_{\mathbf{y}_0} : \mathbb{R}^K \to \mathbb{R}^{K \times K}$ as $\mathrm{M}_{\mathbf{y}_0}(\Theta) := \mathrm{G}_{\mathbf{y}_0}(\Theta)\,\mathrm{G}_{\mathbf{y}_0}(\Theta)^\intercal$, and ii) the scalar-valued function $d_{\mathbf{y}_0} : \mathbb{R}^K \to \mathbb{R}$ as $d_{\mathbf{y}_0}(\Theta) := \det(\mathrm{M}_{\mathbf{y}_0}(\Theta))$. Both are real-analytic in Θ since i) sums and products of real-analytic functions remain analytic, and ii) the determinant of a matrix is a polynomial in its entries.

Now, assume there exists a subset $V \subset \mathbb{R}^K$ for which the entries of $\mathbb{S}(\Theta;\mathbf{y}_0)$, taken as functions of Θ, are not linearly independent for all $\Theta \in V$. Then, by Proposition A3.1 and Lemma A3.1, the GLVM-LSS model is not locally identified for any $\Theta \in V$. Consequently, for all $\Theta \in V$, $\mathrm{G}_{\mathbf{y}_0}(\Theta)$ is rank-deficient, $\mathrm{rank}(\mathrm{M}_{\mathbf{y}_0}(\Theta)) \leq K$, and $d_{\mathbf{y}_0}(\Theta) = 0$ almost surely. We use the following result:

Lemma A3.4 (Corollary A.10 in Gunning and Rossi, 1965)**.** *Let $h : D \to \mathbb{R}$ be a real-analytic function on $D \subseteq \mathbb{R}^K$, and suppose h is not identically zero. Then, the set $V = \{x \in D : h(x) = 0\}$ has Lebesgue measure zero in $\mathbb{R}^K$.*

Proof of Lemma A3.4: Gunning and Rossi (1965), page 9. □

By Lemma A3.4, the set $V = \{\Theta \in \mathbb{R}^K : d_{\mathbf{y}_0}(\Theta) = 0\}$ has Lebesgue measure zero. Thus, the GLVM-LSS model is *not* locally identified only on a set of measure zero. For every $\Theta \in \mathbb{R}^K \setminus V$, where $d_{\mathbf{y}_0}(\Theta) \neq 0$ almost surely, the entries of $\mathbb{S}(\Theta;\mathbf{y}_0)$ are linearly independent for all $\mathbf{y}_0 \in \mathcal{D}$ such that $f(\mathbf{y}_0;\Theta) > 0$ locally. Then, by Proposition A3.1, $\mathcal{I}(\Theta)$ is positive definite, and, by Lemma A3.1, the model is generically locally identifiable in $\mathbb{R}^K \setminus V$. This concludes the proof of Theorem A3.1. ■

In practice, some f_i's might be indexed by distributional parameters that are correlated (yet linearly independent). Due to sampling variability, the latter can lead to situations where the model is not empirically identified. In this case, empirical local iden-

tification of the MLE can be verified if the estimated expected information matrix, $\hat{\mathcal{I}}(\hat{\Theta})$, is non-singular (McDonald and Krane, 1977).

While theorem A3.1 assumes continuous y_i's, establishing general generic local identifiability conditions for discrete y_i's is more challenging due to the finite amount of information in the data. In these cases, parameter identification follows from the existence of a finite-dimensional sufficient statistic. Once appropriate parameter restrictions are imposed, a necessary condition for a GLVM-LSS with categorical items to be identified is that the number of parameters is less than the number of possible response patterns. Let $\Theta_0 \in \mathbb{R}^K$ be a fixed parameter point and A denote the number of possible response patterns (e.g., $A = 2^p$ for binary items or $A = \prod_{i=1}^{p} C_i$ for categorical items, where C_i is the number of categories for item i). Moreover, let $\{\mathbf{y}^{(a)}\}_{a=1}^{A}$ be a finite sequence of observed data points where, for every $a = 1, \ldots, A$, $\mathbf{y}^{(a)} \in \mathbb{N}^p$ satisfies $f(\mathbf{y}^{(a)}; \Theta) > 0$ in an open neighborhood of Θ_0. Following the same logic as above, it suffices to check whether the entries of the score vector $\mathbf{S}(\Theta_0; \mathbf{y}^{(a)})$ are linearly independent.

A4. Empirical Applications: Descriptive statistics

A4a ANES 2020 dataset

Item	Count	Mean	SD	SK	KU
Gay men and Lesbians	7149	0.66	0.27	-0.51	-0.17
Transgender people	7139	0.60	0.28	-0.34	-0.34
Feminists	7159	0.59	0.27	-0.35	-0.40
#MeToo movement	6030	0.59	0.30	-0.45	-0.63
BLM movement	7176	0.53	0.36	-0.26	-1.30
Labor Unions	7148	0.58	0.24	-0.29	-0.09
Journalists	7196	0.51	0.29	-0.26	-0.92
Scientists	7193	0.79	0.20	-1.01	0.94

Table A1: ANES 2020 dataset: Descriptive statistics. *Count*: number of observed responses for each item, *SD*: standard deviation, *SK*: skewness, and *KU*: excess kurtosis.

A4b PISA 2018 dataset

	IRs (y_i's)	log-RTs ($\log(t_i)$'s)			
Item	Prop. correct	Mean	SD	SK	KU
Item 1	0.64	0.19	0.42	0.37	0.83
Item 2	0.41	0.30	0.48	0.79	1.72
Item 3	0.50	0.44	0.62	-0.75	1.58
Item 4	0.36	0.47	0.56	-0.88	2.12
Item 5	0.16	1.00	0.65	-1.05	2.02
Item 6	0.29	0.16	0.52	-0.05	0.95
Item 7	0.06	0.65	0.46	-0.01	1.33
Item 8	0.11	1.02	0.54	-0.65	1.19
Item 9	0.11	0.58	0.51	0.02	0.61

Table A2: PISA 2018 dataset: Descriptive statistics. *Prop. correct*: proportion of students responding correctly to an item; and *SD*: standard deviation, *SK*: skewness, and *KU*: excess kurtosis for the log-RTs.

A5. Simulation Studies: Additional Results

A5a Simulation Study I: Boxplots

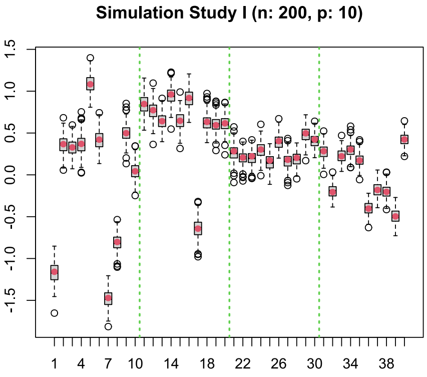

(a) $n = 200$

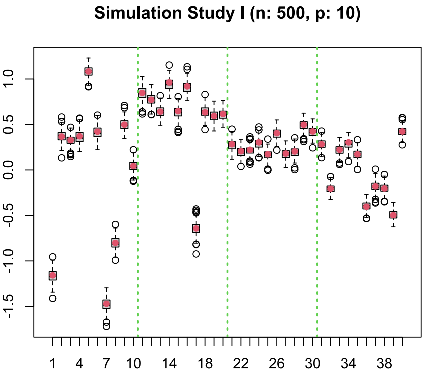

(b) $n = 500$

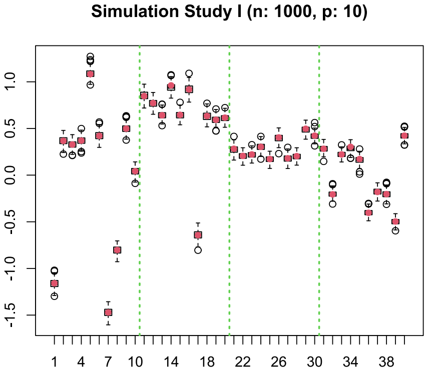

(c) $n = 1000$

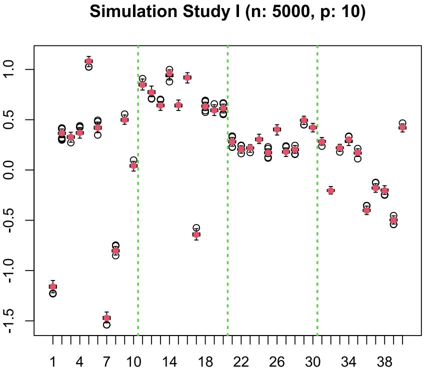

(d) $n = 5000$

Figure A1: Simulation Study I: Boxplots for parameter estimates, $p = 10$. Vertical green lines separate intercepts for the location parameter measurement equation $\alpha_{i0,\mu}$ (first block), factor loadings for the location parameter measurement equation $\alpha_{i1,\mu}$ (second block), intercepts for the scale parameter measurement equation $\alpha_{i0,\sigma}$ (third block), and factor loadings for the scale parameter measurement equation $\alpha_{i1,\sigma}$ (fourth block). Red dots represent true parameter values.

A5b Simulation Study II: Boxplots

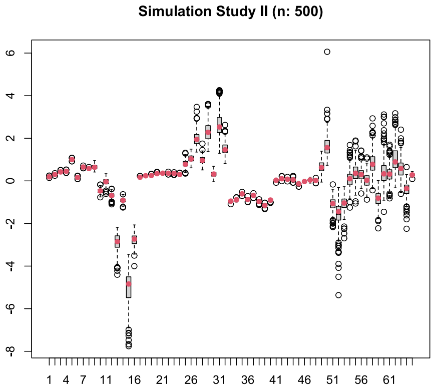

(a) $n = 200$

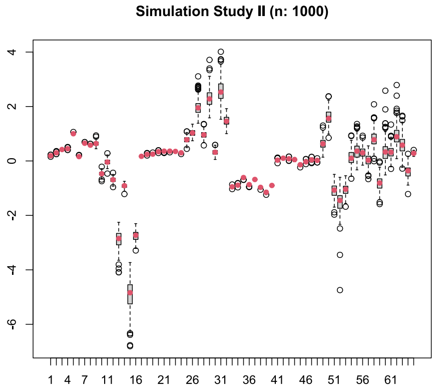

(b) $n = 500$

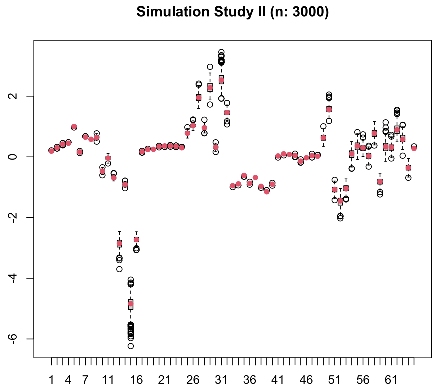

(c) $n = 1000$

Figure A2: Simulation Study II: Boxplots for parameter estimates. First 18 parameters correspond to the intercepts for the location parameter measurement equations ($\alpha_{i0,\pi}$ and $\alpha_{i0,\mu}$). Second group of 18 parameters are the factor loadings for the location parameter measurement equations ($\alpha_{i1,\pi}$ and $\alpha_{i1,\mu}$). Third group of 9 parameters are the intercepts for the scale parameter measurement equation ($\alpha_{i0,\sigma}$). Fourth group of 9 parameters are the factor loadings for the scale parameter measurement equation ($\alpha_{i1,\sigma}$). Fifth group of 9 parameters are the intercepts for the shape parameter measurement equation ($\alpha_{i0,\nu}$). Sixth group of 9 parameters are the factor loadings for the shape parameter measurement equation ($\alpha_{i1,\nu}$). Last parameter is the factor correlation ($\phi_{\mathbf{z}}$). Red dots represent true parameter values.

A5c Additional Simulation Study

We include an additional simulation study to explore how well information criteria (AIC and BIC) help select the true model from two competing alternatives. We generate continuous data in the $(0, 1)$ interval following the location-scale parametrization of the Beta distribution introduced in Appendix A1. The true data generating corresponds to a homoscedastic Beta factor model (Mod0), where only the location parameter depends on a single latent variable, i.e., $y_i \mid z \sim \text{Beta}(\mu_i(z), \sigma_i)$. The competing, more complex alternative is a heteroscedastic Beta factor model (Mod1), similar to that introduced in Section 3.1.

Following Simulation Study I in Section 4.1, the values for the population parameters in the location parameter equation are selected from two uniform distributions: $\alpha_{i0,\mu} \sim \text{Unif}(-1.5, 1.5)$ and $\alpha_{i1,\mu} \sim \text{Unif}(0.5, 1)$. The signs of the $\alpha_{i1,\mu}$'s are assigned randomly with a probability of 0.5. The intercepts for the scale equation are sampled from the uniform distribution $\alpha_{i0,\sigma} \sim \text{Unif}(0.1, 0.5)$. These values ensure the conditional densities $f_i(y_i|z)$ are uni-modal. The integrals involved in parameter computation were numerically evaluated using a fixed-point Gauss-Hermite rule with 100 quadrature points.

We generated $R = 300$ datasets for simulation conditions, combining three different sample sizes (200, 500, and 1000) with three different numbers of observed variables (5, 10, and 20). For each scenario we report the percentage of cases where the AIC or BIC selected Mod1 over Mod0. We also reported the average AIC (AvAIC) and average BIC (AvBIC) for Mod0 and Mod1, respectively. Results are summarized in Table A3 below.

In general, results suggest that almost every time the homoscedastic model (Mod0), the true model, is selected over the competing alternative, the heteroscedastic model (Mod1), when using the AIC or BIC for model selection. More specifically, we observe that using the BIC gives consistent model selection across simulations, as the average number of times that Mod1 is selected over Mod0 by the BIC is zero. The AIC, which focuses on prediction performance (fit to the data), is less accurate for small sample sizes and test length, but it provides better results and both p and n increase.

p	n	Pick Mod1 by AIC (%)	Pick Mod1 by BIC (%)	Mod1		Mod0 (true model)	
				AvAIC	AvBIC	AvAIC	AvBIC
5	200	8.7	0.3	-953.6	-887.7	-958.4	-909.0
	500	9.0	0.0	-2432.9	-2348.6	-2437.8	-2374.6
	1000	7.3	0.0	-4882.0	-4783.9	-4886.9	-4813.3
10	200	2.0	0.0	-2031.6	-1899.7	-2041.7	-1942.8
	500	3.0	0.0	-5128.6	-4960.1	-5138.2	-5011.7
	1000	3.0	0.0	-10326.7	-10130.4	-10336.6	-10189.4
20	200	1.3	0.0	-4527.8	-4264.0	-4547.8	-4349.9
	500	0.3	0.0	-11415.2	-11078.1	-11434.9	-11182.1
	1000	0.3	0.0	-22865.1	-22472.5	-22884.8	-22590.4

Table A3: Additional Simulation Study: Percentage of times the heteroscedastic Beta factor model (Mod1) is selected over the (true) homoscedastic Beta factor model by comparing their AIC and BIC; and average AIC and BIC across simulations for Mod1 and Mod0, by test length and sample size.

References

Allman, E., Matias, C., and Rhodes, J. A. (2009). Identifiability of Parameters in Latent Structure Models with many Observed Variables. *The Annals of Statistics*, 37(6A):3099–3132.

Anderson, T. W. and Rubin, H. (1956). Statistical Inference in Factor Analysis. In Neyman, J., editor, *Proceedings of the Third Berkeley Symposium on Mathematical Statistics and Probability, 1955*, volume V, pages 111–150. University of California Press.

Azzalini, A. (2013). *The Skew-Normal and Related Families.* Institute of Mathematical Statistics monographs, 3. Cambridge, UK: Cambridge University Press, 1st edition.

Bekker, P. A., Merckens, A., and Wansbeek, T. J. (1994). *Identification, Equivalent Models, and Computer Algebra.* Statistical Modeling and Decision Science. San Diego, CA, US: Academic Press.

Bets, L., John, F., and Schechter, M. (1963). *Partial Differential Equations*, volume 3 of *Wiley Series in Lectures in Applied Mathematics.* New York, NY, US: John Wiley & Sons, Ltd, 1st edition.

Browder, F. E. (1961). Real Analytic Functions on Product Spaces and Separate Analyticity. *Canadian Journal of Mathematics*, 13:650–656.

Gu, Y. and Xu, G. (2020). Partial Identifiability of Restricted Latent Class Models. *The Annals of Statistics*, 48(4):2082–2107.

Gunning, R. C. and Rossi, H. (1965). *Analytic Functions of Several Complex Variables.* Englewood Cliffs, NJ, US: Prentice-Hall Inc., 1st edition.

Johnson, B. E. (1969). Separate Continuity and Measurability. *Proceedings of the American Mathematical Society*, 20:420–422.

Krantz, S. G. and Parks, H. R. (2002). *A Primer of Real Analytic Functions.* Birkhäuser Advanced Texts. Boston, MA, US: Birkhäuser, 2nd edition.

Lee, J. D., Sun, Y., and Saunders, M. A. (2014). Proximal newton-type methods for minimizing composite functions. *SIAM Journal on Optimization*, 24.

Lehmann, E. L. and Casella, G. (1998). *Theory of Point Estimation.* Springer Texts in Statistics. New York, NY, US: Springer-Verlag, 2nd edition.

McDonald, R. P. and Krane, W. R. (1977). A note on local identifiability and degrees of freedom in the asymptotic likelihood ratio test. *British Journal of Mathematical and Statistical Psychology*, 30(2):198–203.

Parikh, N. and Boyd, S. (2014). Proximal Algorithms. *Foundations and Trends in Optimization*, 3(1):123–231.

Rigby, R. A., Stasinopoulos, M. D., Heller, G. Z., and De Bastiani, F. (2020). *Distributions for Modeling Location, Scale, and Shape: Using GAMLSS in R.* Chapman & Hall/CRC The R Series. Boca Ratón, FL, US: Chapman & Hall / CRC.

Rothenberg, T. J. (1971). Identification in Parametric Models. *Econometrica*, 39(3):577–591.

Shapiro, A. (1985). Identifiability of Factor Analysis: Some Results and Open Problems. *Linear Algebra and its Applications*, 70:1–7.

Skrondal, A. and Rabe-Hesketh, S. (2004). *Generalized Latent Variable Modeling: multilevel, longitudinal, and structural equation models.* Interdisciplinary Statistics. Boca Ratón, FL, US: Chapman & Hall, CRC.

Zhang, S. and Chen, Y. (2022). Computation for Latent Variable Model Estimation: A Unified Stochastic Proximal Framework. *Psychometrika*, 87(4):1473–1502.
